# Evaluating the chemical exfoliation of lithium cobalt oxide using UV-Vis spectroscopy†

**DOI:** 10.1039/d0na00755b

**Published:** 2020-10-16

**Authors:** Kevin Pachuta, Emily Pentzer, Alp Sehirlioglu

**Affiliations:** Department of Materials Science and Engineering, Case Western Reserve University USA kgp22@case.edu; Department of Materials Science and Engineering, Texas A&M University USA; Department of Chemistry, Texas A&M University USA

## Abstract

Two-dimensional materials have been at the forefront of chemistry and materials science research for the past decade owing to promising applications across many fields. Improvements in exfoliation processes continually give access to new two-dimensional material compositions, demanding a deeper understanding of the defect structure and exfoliation mechanisms. Chemical exfoliation processes allow for both the fabrication of new, and the production of industrial-scale quantities of two-dimensional materials. For this reason, we report a rapid, efficient, and simple method for evaluating the exfoliation behavior of protonated lithium cobalt oxide. Using a two-step chemical exfoliation method, first by proton–cation exchange, then by treatment with an organo-ammonium hydroxide, the exfoliation yield of lithium cobalt oxide was quantified with a quick and powerful technique, ultraviolet-vis spectroscopy characterization. This method provided an in-depth analysis of the exfoliation of lithium cobalt oxide confirming and discovering many key aspects of its soft-chemical exfoliation relating to layered transition metal oxides. It was determined that the exfoliation yield has a strong dependence on multiple factors, such as the concentration of protons in the powder, the presence of water and hydroxide groups in solution, and the ionic radius and concentration of the intercalating cation. Both morphological changes occurring as a function of reaction conditions and the two-dimensional nature of the final sheets were revealed through scanning electron microscopy and atomic force microscopy. Relative proton concentration of acid-treated lithium cobalt oxide was determined as a function of acid concentration using time of flight secondary ion mass spectrometry after deuterated acid treatment. These experiments led to an improved understanding of the soft-chemical exfoliation of lithium cobalt oxide and can be applied to many layered transition metal oxides.

## Abstract 2

The exfoliation yield of protonated lithium cobalt oxide was quantified with ultraviolet-vis spectroscopy. An in-depth analysis using this method confirmed and discovered many key aspects of its soft-chemical exfoliation.

## Graphical abstract

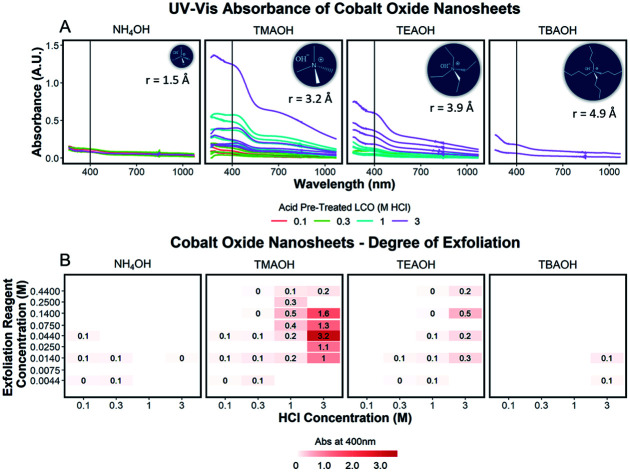

## Introduction

Interest in two-dimensional (2D) materials has grown steadily in the chemistry and materials science communities over the past decade, but in fact, these materials have been utilized for millennia as dyes and other additives.^1^ Notable reports in the scientific literature came as early as 1954 with studies of the swelling and exfoliation of layered micas and clays,^2^ and have extended to present-day for the exfoliation of many other layered materials, such as graphite, layered transition metal dichalcogenides, layered metal oxides, layered double hydroxides, and layered transition metal carbides and nitrides.^1,3–7^ Today 2D materials are commonly produced *via* top-down exfoliation^1,5,6^ and bottom-up synthesis^8,9^ methods in which the starting material is a bulk layered material or atomic precursor, respectively. Top-down exfoliation methods provide the capability for high-throughput, large monolayer sheets and are mainly limited by the size of the bulk crystal.^10^ Generally, layered materials can be classified as either exhibiting interlayer bonding that is van der Waals (graphite, hexagonal boron nitride, black phosphorus, *etc.*) or coulombic (transition metal oxides, transition metal hydroxides, *etc.*). While many layered materials held together *via* van der Waals interactions can be readily mechanically exfoliated *via* the scotch-tape method,^11^ those held together by the stronger interplanar forces of coulombic attraction must undergo chemical treatment to realize their exfoliation. To date, the large-scale production of 2D materials can be a significant limitation in their integration into devices and applications. The underlying mechanisms for producing 2D materials must evolve to develop a deeper understanding as they are developed for next-generation devices and technologies.

With many layered materials available for exfoliation, the properties of 2D materials can often be guided by their layered precursors. For instance, layered materials exhibit a broad range of material properties related to the chemical composition, out-of-plane bonding, in-plane coordination, oxidation state, and doping concentrations.^1^ This wide variety of available material properties gives rise to a substantial amount of selectivity when exfoliating layered materials to form 2D materials for specific applications. Although some properties are inherent to the bulk layered material, many aspects of the material's physical, electronic, and chemical properties may be vastly different for the nanosheet compared to the bulk layered precursor. For example, 2D materials have tremendous anisotropy leading to an exponential increase in surface area-to-volume ratios allowing for efficient catalysts and sensors.^1^ As well, 2D materials exhibit interesting thermal, electronic, and mechanical properties finding applications in transistors, solar cells, batteries, supercapacitors, and more.^1^ Multiple 2D materials can be combined as building blocks to form unique heterostructures creating hybrid ultra-thin devices,^1,5,7^ making 2D materials attractive for many advanced applications.

Chemical exfoliation methods allow for the exfoliation of many layered materials not typically used with traditional mechanical exfoliation methods. These methods, such as the liquid phase intercalation of large alkylammonium cations were first explored for the swelling of layered clays, smectites, and oxides.^12–14^ As early as 1985, the intercalation and exfoliation behavior of layered α-zirconium phosphate was determined to be dependent on the identity and concentration of various intercalating alkylamines.^15,16^ Investigations of protonated layered oxides in the 1990s demonstrated acid–base neutralization reactions in the interlayer and were extended to a variety of layered oxides. This was demonstrated notably with the exfoliation of a layered perovskite,^17^ layered titanate,^18^ layered birnessite,^19^ and layered lithium cobalt oxide (LCO),^20^ among others. These studies led to the discovery of 100-fold swelling of layered titanate giving an improved insight into the intercalation and swelling mechanisms of many layered oxides.^21^ Additionally, recent theoretical studies have increased our understanding of the exfoliation mechanisms of layered materials.^22,23^ Though the processes underlying intercalation, exfoliation, and osmotic swelling are generally understood, quantitative measurements of the exfoliation yield have been elusive. Swelling of protonated layered oxide using multiple intercalates has been explored,^19,21,24–26^ yet only a few studies have related exfoliation yield to ionic size,^25^ with the general acceptance that this process is colligative in nature.^5^ Generally, intercalation effects – which describe the extent of the equilibrium reaction – are quantified through the measurement of intercalated cations in the structure in the bulk (*via* chemical analysis or titration).^19,21^ While these measurements prove useful for osmotic swelling studies, exfoliation yield is not easily deduced from these data. Furthermore, even though the swelling and exfoliation of each layered oxide are generally similar, these materials have slightly different intrinsic properties linked to exfoliation, such as reactivity to acids, acid-exchange potential, interlayer spacing, interlayer cation identity and site occupancy, and inter- and intra-layer bonding. Variations in these intrinsic properties can consequently alter the ability of the layered structure to exfoliate under a given set of conditions, as is the case for LCO.

**Fig. 1:**
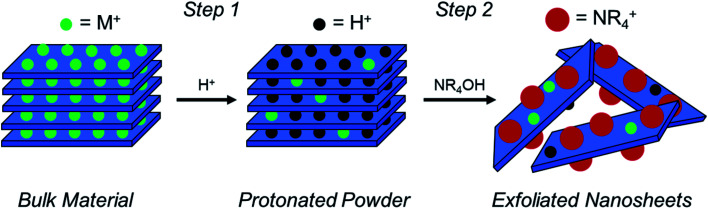
A schematic of the two-step soft-chemical exfoliation process to obtain atomically thin TMO nanosheets from bulk TMOs in which nanosheets are held together by coulombic interactions. Step one uses a protic acid (H^+^) to replace alkali ions with protons in the interstices of the layered structure, producing a “protonated powder”. The second step uses a hydroxide bearing a bulky cation [*i.e.*, a quaternary amine (NR_4_OH)] to promote osmotic swelling and exfoliation of individual TMO nanosheets.

As described in Fig. 1, a layered TMO (typically an alternatively layered structure of transition metal and alkali metal octahedra) can be exfoliated to individual TMO nanosheets using a two-step chemical exfoliation method.^19,20,27,28^ Step one is described by the layered TMO reacting with a protic acid replacing the alkali ions in the interstices of the TMO layers with protons. This can be thought of as two concurrent processes: alkali cation extraction and proton replacement in the interlayer. While alkali cation extraction is incomplete for some layered TMOs, such as LCO, as proven by elemental analysis,^20,27^ acid treatment of commonly exfoliated TMOs such as Cs_*x*_Ti_2−*x*/4_□_*x*/4_O_4_ where *x* = 0.7, Cs_0.8_[Ti_1.2_Fe_0.8_]O_4_, K_0.45_MnO_2_, K[Ca_2_Na_*n*−3_Nb_*n*_O_3*n*−1_], and KCa_2_Nb_3_O_10_ all undergo complete alkali cation extraction as described in Table 1.^17,21,29–31^ However, for layered oxides that undergo complete alkali cation extraction, complete proton replacement does not necessarily always follow. For example, while complete (1 : 1) proton replacement is observed in acid-treated Cs_*x*_Ti_2−*x*/4_□_*x*/4_O_4_ where *x* = 0.7, Cs_0.8_[Ti_1.2_Fe_0.8_]O_4_, K[Ca_2_Na_*n*−3_Nb_*n*_O_3*n*−1_], and KCa_2_Nb_3_O_10_, proton replacement is 0.3 : 1 for K_0.45_MnO_2_.^21,29–31^ Electron transfer between the TMO host layers (*e.g.* MO_2_) forms as a result of this incomplete cation exchange reaction. Changes in valence are most commonly associated with the transition metal^20,27,30^ but are also known to occur for oxygen.^32–34^ Although changes in stoichiometry and valence are occurring, proton replacement is the key driving force for step two, intercalation, exfoliation, and osmotic swelling in the powder.

**Table 1:** The extent of alkali metal extraction and proton replacement, as reported^17,21,27,29–31^

Layered TMO	Alkali metal extraction (%)	Proton replacement (%)	Resulting stoichiometry
LiCoO_2_	63	38	Li_0.37_H_0.24_Co_0.96_□_0.04_O_2_^1.97−^
K_0.45_MnO_2_	100	29	H_0.13_MnO_2_
Cs_*x*_Ti_2−*x*/4_□_*x*/4_O_4_ (*x* = 0.7)	100	100	H_0.7_Ti_1.83_□_0.17_O_4_
Cs_0.8_[Ti_1.2_Fe_0.8_]O_4_	100	100	H_0.8_[Ti_1.2_Fe_0.8_]O_4_
K[Ca_2_Na_*n*−3_Nb_*n*_O_3*n*−1_]	100	100	H[Ca_2_Na_*n*−3_Nb_*n*_O_3*n*−1_]
KCa_2_Nb_3_O_10_	100	100	HCa_2_Nb_3_O_10_

Step two for the exfoliation of TMO nanosheets takes the protonated powder produced after step one and reacts it with a hydroxide bearing bulky cation (typically a quaternary ammonium hydroxide, NR_4_OH, where R = methyl or butyl), where the bulky cation intercalates into the layered host and promotes the swelling and exfoliation of individual TMO layers (*i.e.*, nanosheets). This can be understood first by the reaction of protons located in the interlayer with hydroxide (OH^−^) groups dissociated from the bulky organo-ammonium cations in solution, which produces water. Osmotic pressure and charge balancing of the structure results in an influx of water and bulky cations into the interlayer, thereby promoting swelling of the structure and exfoliation of the TMO layers. Osmotic pressure in the solution is subsequently governed by the [NR_4_^+^]/[H^+^] ratio in the reaction, where proton concentration is the concentration of protons in the powder and NR_4_^+^ concentration is the concentration of bulky cations in solution.^5,20,21,28,30,35^ Previous reports, including the exfoliation of LCO, used only one proton concentration. Therefore, the dependence on proton concentration in the system, and consequently the [NR_4_^+^]/[H^+^] ratio, have not been fully revealed. Furthermore, proton concentration determined *via* indirect measurements such as iodometric titration and thermogravimetric analysis (TGA) may allow for discrepancies in the true proton concentration in the powder as compared to the reported values. Therefore, changes in alkali cation and proton concentration in the powder (*i.e.*, different acid treatments) may have considerable effects on the ability for TMOs to swell and exfoliate.

Here we report a rapid, efficient, and simple method for evaluating the exfoliation yield of TMOs using UV-Vis spectroscopy, using the preparation of cobalt oxide nanosheets (CONs) from bulk LCO powder as an example. Due to the inherent absorption profiles of numerous 2D materials, UV-Vis spectroscopy can be implemented as a way to observe exfoliation, and may also provide insight into the intrinsic properties of the exfoliated material, such as the band-gap, solvent effects, surface functional groups, lateral size, thickness, vacancies, *etc.* Using UV-Vis absorbance as the defining characterization technique for exfoliation, we performed a systematic study of multiple exfoliation conditions (acid concentration, tetraalkylammonium identity, and tetraalkylammonium concentration) to establish their impact on exfoliation yield. Combining these results with measured relative proton concentration values of multiple acid-treated LCO powders, exfoliation of LCO was examined as a function of relative proton concentration, the cation radius of NR_4_OH, and concentration of reagents. The wide range of reaction conditions investigated for the exfoliation of LCO can be used as a guide for the exfoliation of other layered oxides. Specifically, fundamental relationships between the layered host and the exfoliation conditions are confirmed updating known mechanisms for the two-step soft-chemical exfoliation of LCO and other layered TMOs.

## Experimental

### Reaction conditions

**Fig. 2:**
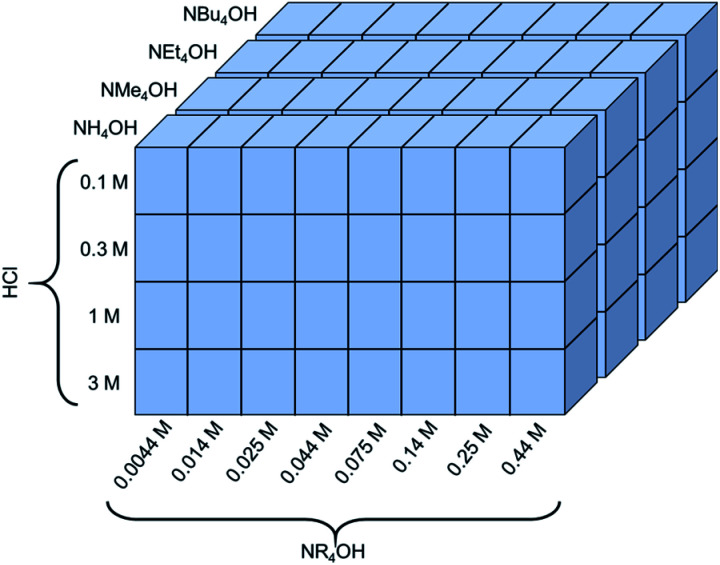
A schematic of the experimental space used to investigate the dependence of proton concentration, NR_4_OH concentration, and NR_4_^+^ ionic radius on the exfoliation yield of LCO.

The exfoliation of LCO was studied using various two-step soft-chemical reactions. Step one, separate batches of LCO (MTI corp.) were treated with hydrochloric acid (HCl) (36.5–38 wt%, Fisher Scientific) at four different concentrations (0.1 M, 0.3 M, 1 M, and 3 M HCl). The reaction proceeded for 24 hours with 10 mg LCO per mL HCl at room temperature and pressure in a sealed round bottom flask (RBF) mixing at 400 rpm. Each of these powders was washed with copious amounts of deionized water to remove any remaining acid and salts formed from the reaction. Each powder was allowed to dry in air overnight then stored in a capped glass vial until its use. In step two, the acid-treated powders were treated separately at four initial concentrations of tetraalkylammonium hydroxide aqueous solutions (0.014 M, 0.044 M, 0.14 M, and 0.44 M NR_4_OH, where R = H, methyl, ethyl, butyl). These exfoliation reagents studied are ammonium hydroxide (28.4 wt%, Fisher Sci.), tetramethylammonium hydroxide (25 wt%, Alfa Aesar), tetraethylammonium hydroxide (35 wt%, Alfa Aesar), and tetrabutylammonium hydroxide (40 wt%, Alfa Aesar). Additional concentrations at 0.0044 M NR_4_OH, 0.025 M NR_4_OH, 0.075 M NR_4_OH, 0.25 M NR_4_OH were tested for certain acid-treated powders if exfoliation was expected in these regions of interest. Due to the inherent etching nature of the exfoliation reagents chosen, plastic containers were used to prevent the dissolution of the glassware (dissolution of cations from the glassware into the solution will cause the colloidal suspension of CONs to agglomerate). As a result, these powders were reacted for 24 hours in plastic centrifuge tubes at 10 mg protonated LCO per mL NR_4_OH under room temperature and pressure with vigorous stirring (1000 rpm). After the reaction was complete the solutions were centrifuged for a total of 20 minutes at 4500 relative centrifugal force (rcf) (or 5000 rpm using an Eppendorf A5810). In total, 95 experimental exfoliation conditions were examined using UV-Vis spectroscopy, spanning a wide range of HCl (0.1–3 M, 30×) and NR_4_OH (0.0044–0.44 M, 100×) concentrations, as summarized in Fig. 2. Additional reactions were completed in water, tetramethylammonium chloride (synthesized through acid–base neutralization of TMAOH and HCl), and TMAOH in methanol (25 wt%, Arcos Organics).

### Characterization

To analyze the effective concentration of 2D CONs exfoliated *via* each reaction condition, UV-Vis spectroscopy was utilized to measure the absorbance of each sample after centrifugation. By capitalizing on the intrinsic absorbance of suspended 2D CONs in an aqueous solution, the exfoliation yield from each reaction condition was established. Absorbance measurements of each sample were completed using an integrating sphere on a Cary 6000i UV-Vis-NIR Spectrophotometer. Using this experimental set-up, any scattering of light due to the presence of larger particles was collected by the detector, thus, removing scattering artifacts from these measurements. Absorbance data were collected from 1075 nm to 260 nm and the absorbance of each reagent solution the nanosheets were suspended in was subtracted. Any absorbance below 260 nm was ignored due to reagent absorption in this region (ESI Section Spectra analysis, Fig. S1†). Samples with high concentrations of dispersed CONs were diluted such that the measured absorbance was less than 1.5 A.U. to avoid saturation of the detector. The recorded UV-Vis data were processed and analyzed with R.^36^ To compare exfoliation yield for multiple reaction conditions, the absorbance of each diluted sample was scaled according to their dilution factor. The absorbance for each sample at 400 nm was used to determine the relative exfoliation under each condition after a background value was subtracted. This background value was due to the absorbance of unexfoliated LCO particles suspended in solution (Fig. S3†). To properly subtract the baseline absorbance across the entire spectrum, the absorbance at 1075 nm was used due to negligible absorption from the exfoliated CONs in this region. Optical images of selected reactions were taken and are shown in the ESI Section Spectra analysis, Fig. S2.† UV-Vis measurements of solutions of CONs were completed after 30 and 60 days of storage in sealed centrifuge tubes (ambient temperature and pressure). To prevent the measurement of loose sedimentation as a result of aging, aged CONs samples were centrifuged for 5 minutes at 4200 rcf before transferring to a cleaned UV-Vis cuvette. UV-Vis absorbance was measured for as received LCO powder using the same instrument set-up. To prevent sedimentation, LCO powder was suspended in a solution of polyethylene glycol; a blank solution of PEG was subtracted from the measurement.

The presence, thickness, and lateral dimensions of the exfoliated nanosheets were confirmed using atomic force microscopy (AFM) (Park Nanosystems NX10) on selected samples after the solution was drop-cast onto freshly cleaved mica substrates. To visualize the powders before and after each reaction (isolated pellet from centrifugation), scanning electron microscopy (SEM) (FEI Helios Nanolab 650) was utilized. To determine the lattice parameters of the powders after HCl treatment, X-ray diffraction (XRD) (Bruker D8 Advance) was completed. Time of Flight Secondary Ion-Mass Spectroscopy (ToF-SIMS) (ToF.SIMS 5, IONTOF) was used to determine the concentration of protons in the interlayer structure of LCO (more details in ESI Section Acid treated powders, Fig. S10†). This was accomplished by measuring deuterium intensity in LCO powders after reaction with 0.1 M, 0.3 M, 1 M, and 3 M deuterium chloride (diluted in deuterium oxide) solutions. Reactions were carried out using the same reaction parameters as before (10 mg LCO per mL acid solution, RBF, constant stirring) but were kept in under inert atmosphere during the reaction and in storage (N_2_ purge, to prevent deuterium–proton exchange). To analyze the bulk concentrations of elemental species in the treated LCO powders, inductively coupled plasma mass spectroscopy (ICP-MS) was completed using a PerkinElmer NexION 300D.

## Results and discussion

### Exfoliation of LiCoO_2_ (LCO) using “standard” conditions

**Fig. 3:**
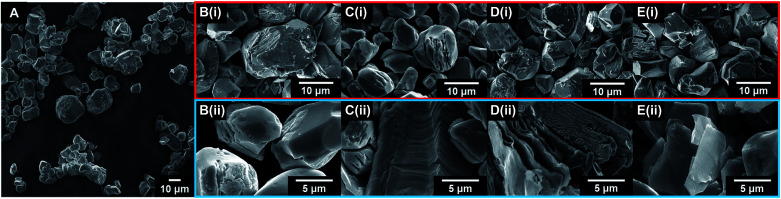
SEM micrographs of as-received and treated LCO powders. Selected powders shown are (A) LCO as-received, (B(i)) 0.1 M HCl treated LCO, (B(ii)) 0.1 M HCl + 0.014 M NH_4_OH treated LCO, (C(i)) 0.3 M HCl treated LCO, (C(ii)) 0.3 M HCl + 0.014 M NH_4_OH treated LCO, (D(i)) 1 M HCl treated LCO, (D(ii)) 1 M HCl + 0.14 M TMAOH treated LCO, (E(i)) 3 M HCl treated LCO, (E(ii)) 3 M HCl + 0.044 M TMAOH treated LCO. All powders were reacted for 1 day under constant stirring. SEM micrographs highlighted in red are after acid treatment (step one). SEM micrographs highlighted in blue are after acid and amine treatment (step two).

The exfoliation of LCO was accomplished using a two-step wet-chemical exfoliation method first reported by Kim *et al.*^20^ and improved in our recent work.^27,37^ After the second step of the reaction was completed, centrifugation of the resulting slurry allowed for the separation of larger particles (pellet) from colloidally stabilized nanosheet suspensions (supernatant). SEM characterization of the centrifuged pellet showed significant morphological changes under known exfoliation conditions with tetramethylammonium hydroxide (TMAOH) (*i.e.* 3 M HCl and 0.044 M TMAOH treatment, 1 M HCl and 0.14 M TMAOH treatment). For instance, swelling and plasticity of the layered structure occurred as compared to LCO powder Fig. 3A, and acid-treated LCO Fig. 3B(i)–E(i). This is attributed to the influence of intercalated bulky cations in between the layers of cobalt-oxide octahedra (Fig. 3D(ii) and E(ii)). Other reaction conditions, such as reaction with ammonium hydroxide (NH_4_OH), displayed no significant changes in structure or morphology (Fig. 3B(i and ii)). For a given concentration of TMAOH, the most drastic changes were observed for powders that were exposed to higher concentration acid in step one. For example, as shown in Fig. 3D(ii), swelling of 1 M HCl treated powder with TMAOH led to the substantial separation of the layered structure. Fig. 3E(ii) demonstrates the complete exfoliation of large plate-like particles from LCO and is indicative of the importance of using the appropriate acid-treatment conditions en route to the exfoliation of LCO and layered TMOs.

**Fig. 4:**
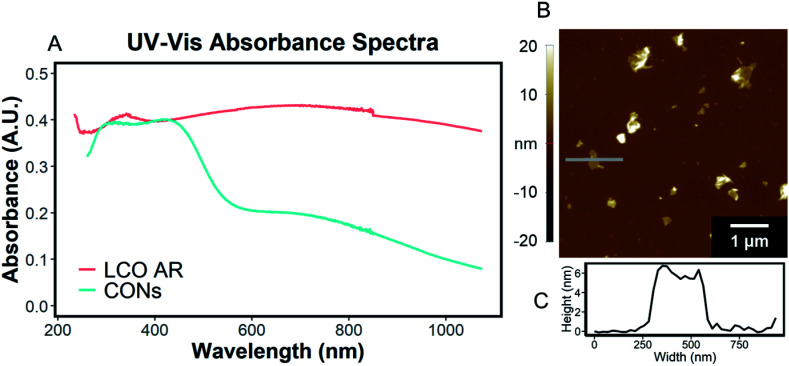
(A) UV-Vis absorbance spectra of a solution of CONs (blue) and lithium cobalt oxide powder (orange). CONs (prepared from 1 day 3 M HCl and 1 day 0.044 M TMAOH treatment) were suspended in 0.044 M TMAOH and were separated from bulk powder *via* centrifugation. Lithium cobalt oxide was suspended in polyethylene glycol to prevent sedimentation. (B) AFM image and (C) line scan height profile of the same solution of CONs drop cast onto a clean mica substrate.

UV-Vis characterization of the supernatant shows the characteristic absorption profile of the exfoliated CONs dispersed in solution. Fig. 4A shows the difference between the absorption of LCO powder, and exfoliated CONs dispersed in solution. A broad featureless absorption was observed for LCO powder, while three distinct absorbance bands were observed for the supernatant. The stark difference in absorption profiles between these two materials is due to the presence of 2D CONs in solution, as verified using AFM (Fig. 4B). The broad nature of the absorption peaks of the nanosheets indicates a distribution of thicknesses and lateral sizes. It is assumed that any absorbance is due to 2D CONs in solution and not remaining bulk particles in solution. Currently, these data cannot provide quantitative statistical representations of the particle size, shape, thickness, or defect concentration, but these analyses are currently under investigation in our lab.

### Impact of acid treatment of LCO

The first step in the exfoliation procedure is completed *via* acid treatment of LCO with aqueous HCl (Fig. 3B(i)–E(i)). As discussed in previous work, the determination of lithium, cobalt, and oxygen content in the treated powder can be quite difficult and has been reported for only one treatment condition (1 M HCl treated LCO).^27^ Unless neutron diffraction is readily available, the most meaningful way to report the lithium content in the sample is through the Li/Co ratio of the powder *via* inductively coupled plasma-mass spectrometry (ICP-MS) analysis. However, stoichiometry composition based on ICP-MS characterization can be misleading due to cobalt vacancies derived from acid treatment,^27^ known stability of lithium and cobalt intermixing, and oxygen vacancies,^38^ or changes in bonding iconicity after delithiation.^32–34^ For the acid-treated powders investigated, the Li/Co ratio decreased logarithmically as a function of HCl concentration, as determined by ICP-MS (Table 2 and ESI Section Acid treated powders, Fig. S8†). However, at concentrated HCl solutions (∼12 M), LCO completely dissolved after 24 hours. Increasing the time for acid treatment in each diluted condition increased the Li removal only asymptotically, reaching at or near the limit at the times considered.^39^ Thus, competing effects of dissolution and ionic replacement associated with acid treatment limit the full ionic replacement of Li-ions with protons in LCO. Therefore, the acid treatment of LCO must be tuned appropriately to achieve high degrees of proton replacement together with a maximal yield of protonated powder (*i.e.*, to prevent the dissolution of the powder and resulting nanosheets). Due to the drastic stoichiometric changes occurring in the powder, XRD was utilized to determine if any crystallographic changes occurred as a result of acid treatment. No major changes in structural symmetry are reported, but an increase in the *c*-lattice parameter was observed for each acid treatment (XRD plots are shown in ESI Section Acid treated powders, Fig. S9† and Table 2) and is attributed to a decreased coulombic attraction between the cobalt oxide layers and the interstitial Li-ion. This suggests weaker bonding between protons and cobalt oxide sheets. The minor decrease in the *a*-lattice parameter upon treatment with more concentrated acid is attributed to the tighter bonding between cobalt and oxygen in the in-plane (orthogonal to 001) direction and relates to changing iconicity of cobalt–oxygen bonding.

**Table 2:** Li/Co ratio (ICP-MS), and the *a*- and *c*-lattice spacing (XRD and Rietveld refinement) are shown for LCO as received, 0.1 M HCl treated, 0.3 M HCl treated, 1 M HCl treated, and 3 M HCl treated. D/Co (ToF-SIMS) data are shown for LCO as received, 0.1 M DCl treated, 0.3 M DCl treated, 1 M DCl treated, and 3 M DCl treated. All powders were reacted for 1 day under constant stirring

LiCoO_2_ treatment	Li/Co ICP-MS	*a*-Lattice (Å) XRD	*c*-Lattice (Å) XRD	D/Co ToF-SIMS^a^
As received	1.04	2.819	14.07	—
0.1 M HCl	0.77	2.813	14.23	1.37
0.3 M HCl	0.56	2.811	14.38	0.85
1 M HCl	0.42	2.809	14.38	2.12
3 M HCl	0.37	2.812	14.40	7.42

a Deuterium powder samples. The measured D/Co ratios in the powder are greater than 1, therefore not permitted through charge balance in the powder, the sensitivity of deuterium may need to be corrected for absolute concentrations to be determined.

Proton replacement was further investigated by ToF-SIMS analysis of LCO treated with 0.1 M, 0.3 M, 1 M, and 3 M DCl in D_2_O (referred to as deuterated power samples). The relative concentrations of deuterium (*i.e.*, protons) examined as a function of DCl treatment were compared with the Li/Co ratios determined by ICP-MS. Measurements of pristine LCO powder showed very low deuterium intensity, attributed to noise within the measurement; this value was subtracted as a baseline from each acid-treated sample. Analysis of each fragment was completed using MCs^+^ and MCs_2_^+^ clusters (where M = matrix components ^2^H, ^7^Li, and ^59^Co) to reduce the matrix and surface effects typically encountered in ToF-SIMS measurements when pure matrix fragments are used.^40–43^ Since the Li/Co ratio is known for each sample from ICP-MS analysis, sensitivity factors were used to correct the measured CsLi^+^/CsCo^+^ ratios of each sample. As shown in Table 2, deuterium (D) – which is assumed to have the same chemical potential and undergo the same reactions with LCO as protons – was measured at significantly higher intensities in acid-treated powders compared to untreated LCO. Relative deuterium concentration was compared for each sample by monitoring the D/Co ratio. At higher acid concentrations, higher D/Co ratios were measured, directly proving the presence of protons in the interlayer. For instance, the measured D/Co ratio for 3 M DCl treated LCO is approximately three times that of 0.1 M DCl, proving that increased concentration of acid treatment imparts both more alkali cation extraction (ICP-MS) and proton replacement (ToF-SIMS) in the powder. Normalized ToF-SIMS plots for each matrix component compared to total counts for each sample are shown in ESI Section Acid treated powders, Fig. S10† with a full table and description of the calculations in ESI Section Acid treated powders, Tables S1 and S2.† As reported for the acid-treatment of layered TMOs, hydrated H^+^ (*i.e.*, hydronium ions) could be present in the interlayer or as surface adsorbates after acid treatment.^12,21^ To mitigate these effects, the treated powder was dried and stored under N_2_ to eliminate adsorbed ambient water. Additionally, XRD measurements of LCO and acid-treated LCO suggest the presence of water in the interlayer is minimal. Since the diameter of a water molecule (2.75 Å) is more than half the interlayer spacing (4.68 Å, 001 direction *d*-spacing) of untreated LCO, interlayer water molecules are expected to cause a substantial increase in the measured (003) *d*-spacing, as observed with HCa_2_Nb_3_O_10_.^31^ It was found that the interlayer expansion of LCO is only ∼3% after treatment with even the highest acid concentration, interlayer hydration is likely not occurring. Additional XRD measurements of wet acid-treated LCO stored in DI water (to prevent any drying, and therefore expulsion of water from interlayer) show no significant changes in lattice parameters, again confirming that the interstices of the layered structure after acid treatment do not contain water molecules (ESI Section Acid treated powders, Table S3†).

### UV-Vis spectroscopic analysis of reaction solutions

**Fig. 5:**
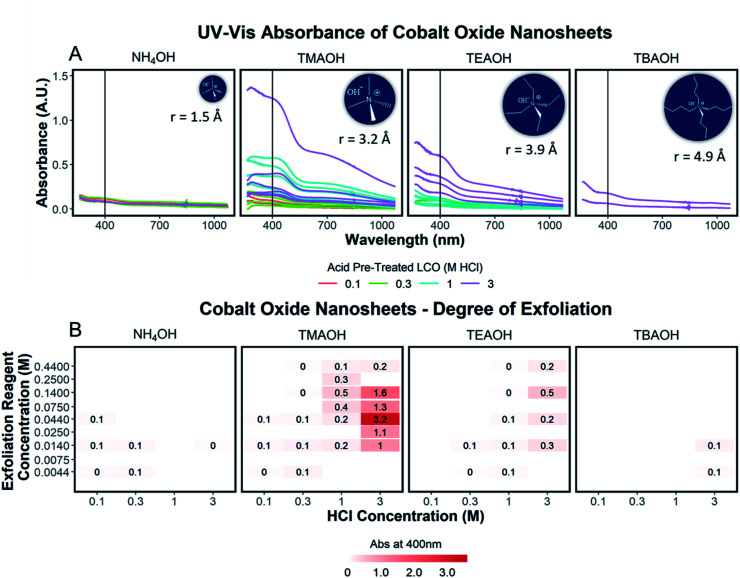
(A) UV-Vis absorbance spectra of solutions of exfoliated CONs organized by the ionic radius of the exfoliation reagent used, increasing left to right. The color of each spectrum represents the concentration of the acid used in step one, signifying varying degrees of proton replacement. A vertical line at 400 nm indicates the absorbance used to determine the degree of exfoliation. (B) The degree of exfoliation derived from the spectra in (A) plotted in a 2D array with pre-treated acid powder (0.1 M HCl, 0.3 M HCl, 1 M HCl, 3 M HCl) as the *x*-axis, the concentration of the exfoliation reagent (0.0044 M, 0.0075 M, 0.014 M, 0.025 M, 0.044 M, 0.075 M, 0.14 M, 0.25 M, 0.44 M) as the *y*-axis, and separated by the ionic radius of the exfoliation reagent used, increasing left to right. A color scale at the bottom of the plot illustrates the variation of color corresponding to the degree of exfoliation. Adjusted absorbance is shown for the degree of exfoliation and accounts for sample dilution by multiplying measured absorbance by the value of the dilution. All raw data, individual spectra analysis, and R code is published in an OSF project https://osf.io/xkme6/ (DOI: 10.17605/OSF.IO/XKME6).

To quantify the effects of and relationship between step one and step two, the impact of proton concentration, NR_4_^+^ concentration, and NR_4_^+^ ionic size on the exfoliation of LCO was evaluated by UV-Vis absorbance spectroscopy. Specifically, the supernatant obtained after step two of the reaction was characterized. The exfoliation yield of each reaction condition was able to be determined using the fundamental relationship between measured absorbance and concentration (Beer–Lambert law). In other words, the measured absorbance from each reaction condition was directly related to the concentration of CONs in solution. Just under half of the reactions exhibited characteristics relating to the presence of 2D CONs as described by three broad absorbance peaks in the UV-Vis region, while the others showed no peaks and therefore indicated a lack of exfoliation. For more information regarding the absorbance spectra of all samples, refer to ESI Section Spectra analysis, Fig. S2.† For samples containing 2D CONs, the absorbance at 400 nm was determined to be the most reliable wavelength (when compared against 270 nm and 700 nm) for consistency as a function of concentration with no overlapping solvent peaks (water, NR_4_OH) (ESI Section Spectra analysis, Fig. S1 and S3†). As shown in Fig. 5A, this wavelength is indicated by the vertical line on each spectrum, and Fig. 5B plots the absorbance at 400 nm for each reaction as a function of acid concentration (*x*-axis), and NR_4_^+^ concentration (*y*-axis) for different NR_4_^+^ sizes (each panel) to elucidate trends in LCO exfoliation behavior.

### Significance of interlayer protons

A significant dependence of the exfoliation yield on proton concentration in the protonated layered oxide is quantitatively shown in Fig. 5B. In short, the conditions used to treat LCO with protic acid (step 1) directly impact the conditions of step 2 needed for exfoliation. Exfoliation is not as prevalent in the protonated powders tested with low proton concentration (0.1 M HCl and 0.3 M HCl treated LCO), regardless of the NR_4_^+^ ionic size or concentration. For a given cation, increasing proton concentration increased the exfoliation yield. When the maximum exfoliation yield achievable is high (*e.g.*, 3 M HCl and 0.044 M TMAOH treatment), exfoliation yield can be achieved across many NR_4_^+^ concentrations (*e.g.*, high exfoliation yield 3 M HCl treated LCO from 0.014–0.14 M TMAOH). While high [H^+^] in the protonated powder is ideal to achieve the highest exfoliation yield and is possible for many other layered TMOs such as Cs_*x*_Ti_2−*x*/4_□_*x*/4_O_4_ (*x* = 0.7) K_0.45_MnO_2_, K[Ca_2_Na_*n*−3_Nb_*n*_O_3*n*−1_], and KCa_2_Nb_3_O_10_, the solubility of LCO in acidic solutions creates an upper limit on the [H^+^] that can be obtained. Therefore, for TMOs with less stability towards acid solutions, it is crucial to determine a suitable treatment method that allows for high alkali ion removal and high proton replacement, while minimizing the amount of dissolution occurring. To illustrate this point, reaction conditions with TMAOH, tetraethylammonium hydroxide (TEAOH), and tetrabutylammonium hydroxide (TBAOH) all showed increased exfoliation yield as the concentration of acid treatment increased. Reaction with NH_4_OH solutions proved insignificant for the exfoliation of protonated LCO. For this study, it was determined that 3 M HCl treatment provided the best conditions for step 1, providing a broader range of concentrations useful for exfoliation in step 2.

### Significance of water and hydroxide ions in solution

The reliance of proton concentration in the powder for swelling and exfoliation confirms the two-step chemical reactions in the interlayer as suggested by others.^5,21^ To test this hypothesis, known reaction conditions promoting exfoliation (step 1 = 3 M HCl; step 2 = 0.044 TMAOH), were mimicked with tetramethylammonium chloride (TMACl), a salt with a non-basic anion. The reaction of protonated LCO with TMACl under these conditions produced a clear and colorless supernatant after centrifugation of the reacted slurry, indicating that no exfoliated occurred and confirming that a Brønsted base (OH^−^) is required for exfoliation.^21^ To determine if an aqueous reaction environment is needed to supply the influx of water in the interlayer and promote swelling and exfoliation, a methanolic solution of TMAOH was used in place of the aqueous solution. As with the TMACl conditions, a clear and colorless supernatant was produced after centrifugation of the reaction slurry, again indicating no exfoliation occurred. The size of methanol is larger than water and has methanol has a lower polarity and dielectric constant, which may prevent its diffusion to the interlayer. These observations support that the neutralization of H^+^ with OH^−^ to form water and an aqueous environment is essential for the introduction of bulky cations and the promotion of exfoliation. Images and spectra of these reactions are shown in ESI Section Spectra analysis, Fig. S3.†

### Significance of NR_4_^+^ concentration

Relationships between the NR_4_^+^ concentration in solution and proton concentration in the protonated layered TMO were investigated. As shown in Fig. 5B, the exfoliation yield of step two has a strong dependence on the concentration of the exfoliation reagent used in the reaction. For instance, TMAOH, TEAOH, and TBAOH all showed an increase in exfoliation yield at selective NR_4_^+^ concentration when varying proton concentration in protonated LCO. This means for a specific NR_4_^+^ size and proton concentration, the exfoliation yield is maximized at a specific NR_4_^+^ concentration. This reveals a [NR_4_^+^]/[H^+^] ratio (dependent on the NR_4_^+^ size) that is suitable for exfoliation, as commonly described in the literature. Although, as is the case with TMAOH, the [NR_4_^+^]/[H^+^] relationship does not hold across multiple proton concentrations. In other words, if the [NR_4_^+^]/[H^+^] ratio was a true indication of the exfoliation yield, lower proton concentration in protonated LCO would require lower NR_4_^+^ concentration to promote exfoliation. Alternatively, higher proton concentration in protonated LCO would require higher NR_4_^+^ concentration to promote exfoliation. However, our results showed that the maximum exfoliation yield was obtained at the following conditions 0.1/0.014 M HCl/TMAOH, 0.3/0.014 M HCl/TMAOH, 1/0.14 M HCl/TMAOH, 3/0.044 M HCl/TMAOH. Thus for protonated LCO, the [NR_4_^+^]/[H^+^] ratio is not a good indicator of optimum exfoliation conditions since proton concentration may vary within for various acid-treatment conditions. This deviation from the preferred [NR_4_^+^]/[H^+^] ratio may be a result of the changing concentrations of both Li^+^ and H^+^ in the powder as well as other chemical or bonding changes in the material as a result of different acid treatments.

### Significance of NR_4_^+^ ionic size

The ionic size of the exfoliation reagent is yet another crucial factor in the success of the exfoliation reaction. As shown in Fig. 5B, TMAOH (*r* = 3.2 Å) provided the best yield of exfoliation for every protonated LCO tested, with an order of magnitude increase in the yield of exfoliated material in comparison to NH_4_OH, TEAOH, and TBAOH. The results shown in Fig. 5B confirm that specific reaction concentrations dictate the swelling and subsequent exfoliation of protonated LCO, but do not coincide with results of exfoliation for multiple exfoliation reagents. H_0.8_[Ti_1.2_Fe_0.8_]O_4_, H_*x*_Ti_2−*x*/4_□_*x*/4_O_4_ (*x* = 0.7), H_0.13_MnO_2_, and HCa_2_Nb_3_O_10_ have been reported to be exfoliated using reagents with large differences in cation size, namely TMAOH and TBAOH (0.64 nm and 0.98 nm respectively, TBA^+^ is 1.5× bigger than TMA^+^).^19,21,24–26^ The UV-Vis data (Fig. 5B) demonstrates that protonated LCO does not exhibit similar swelling properties compared to these other layered oxides. If this was the case, exfoliation of protonated LCO with TMAOH and TEAOH would be expected to display similar exfoliation yield, since both cations have a similar ionic diameter (0.64 nm and 0.78 nm respectively, TEA^+^ is 1.2× bigger than TMA^+^). Reactions of protonated LCO with TMAOH provide a significantly greater exfoliation yield compared to reactions with TEAOH, confirming that protonated LCO exhibits different exfoliation behavior than other layered oxides, contrary to previous reports.^5^ These findings elucidated a key aspect of exfoliation of protonated LCO which is often overlooked with other layered oxides – interlayer spacing. By examining the interlayer spacing of LCO after acid treatment and comparing it to the ionic diameter of each reagent used in the exfoliation reaction, a critical diameter of the cation relative to the interlayer spacing is proposed for the exfoliation of protonated LCO. For ionic diameters that were considerably small (NH_4_^+^) compared to the interlayer spacing of LCO, exfoliation did not proceed. Alternatively, for ionic diameters much larger (TBA^+^) than the interlayer spacing of LCO, exfoliation also did not proceed. The ionic diameter of the exfoliation reagent that proved most effective for LCO was approximately 1.3× the size of the interlayer spacing. Other layered oxides such as H_0.8_[Ti_1.2_Fe_0.8_]O_4_, H_*x*_Ti_2−*x*/4_□_*x*/4_O_4_ (*x* = 0.7), H_0.13_MnO_2_, H[Ca_2_Na_*n*−3_Nb_*n*_O_3*n*−1_], and HCa_2_Nb_3_O_10_ have much larger interlayer spacing than protonated LCO (0.89,^21^ 0.94,^18^ 0.73,^30^ 1.6–2.8,^14^ 1.44,^31^ and 0.48 (ref. 20 and 27) nm, respectively), possibly reducing the effects the ionic diameter has in relation to the interlayer spacing. For example, the interplanar spacing in other protonated oxides is large enough that water (diameter of 0.28 nm) and bulky cations can easily diffuse into the layered host promoting exfoliation. Conversely, for more compact layered oxides, such as LCO, the diffusion of bulky cations and water molecules may not be as favorable, thereby imposing stricter limits on the ionic size needed for exfoliation in relation to the interlayer spacing. Furthermore, the intercalation of these materials with smaller cations such as Na^+^, K^+^, or NH_4_^+^ may not lead to a significant enough expansion of the layers to promote water intercalation, swelling, and exfoliation.

### Effects of aging

The exfoliation yield of protonated LCO was determined to be dependent on the aging time between step 1 and step 2. After the acid-treated powder was washed with DI water and dried overnight in air, the powder was stored in a capped glass vial. This powder was reacted using the identical conditions as before (step 1 = 3 M HCl; step 2 = 0.044 M TMAOH), but after 25 and 150 days of storage in the glass vial. As shown in ESI Section Spectra analysis, Fig. S5† the UV-Vis absorbance of the supernatant collected after 1 day of reaction and therefore exfoliated CONs in solution is reduced by over half (−56%) after 25 days in storage and by three quarters after 150 days (−75%). Though the effects of powder aging corresponding to exfoliation yield are drastic, the mechanism for aging is currently not clear. XRD measurements (ESI Section Acid treated powders, Table S3†) do not show supporting evidence for changes associated with aging. Very small changes in the lattice spacing were observed (−0.1% for the *c*-lattice parameter) for the powder measured after approximately the same period (167 days) meaning no significant bonding or structural changes occurred within this time frame. It can be speculated that the decrease in efficiency by aging may be due to drying or redistribution of Li^+^ and H^+^ in the powder over time. This mechanism is suggested as the protonation reaction starts at the surface and proceeds into the powder, and it has been shown that Li is remaining in the powders after this first step. However, currently, we do not have a direct observation of changes in ion distributions with aging time. Additional exfoliation parameters were considered such as the time of the exfoliation reaction, protonated LCO loading, and stirring rate and presented in ESI Section Spectra analysis, Fig. S5.†

**Fig. 6:**
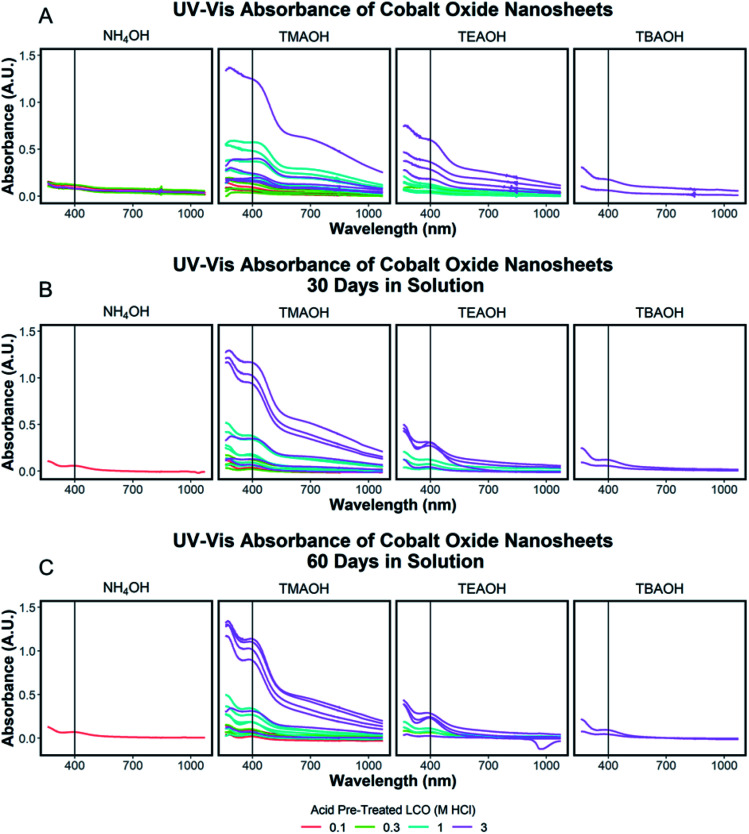
(A–C) UV-Vis absorbance spectra of solutions of exfoliated CONs separated by the ionic radius of the exfoliation reagent used. The color of each spectrum represents the concentration of the acid of pre-treated LCO signifying varying degrees of proton replacement in the structure. A vertical line at 400 nm indicates this wavelength was taken as the value for the degree of exfoliation. Spectra are shown for as-prepared samples (A), after 30 days (B), and after 60 days (C).

**Fig. 7:**
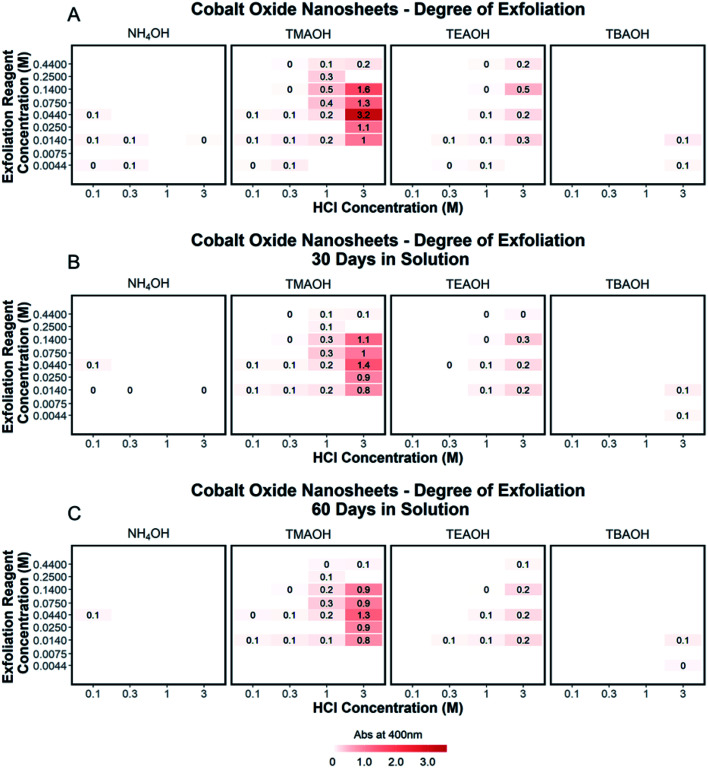
(A–C) The degree of exfoliation for each spectrum was plotted in a 2D array with pre-treated acid powder (0.1 M HCl, 0.3 M HCl, 1 M HCl, 3 M HCl) as the *x*-axis, the concentration of the exfoliation reagent (0.0044 M, 0.0075 M, 0.014 M, 0.025 M, 0.044 M, 0.075 M, 0.14 M, 0.25 M, 0.44 M) as the *y*-axis, and organized by the ionic radius of the exfoliation reagent used. A color scale at the bottom of the plot shows the variation of color corresponding to the degree of exfoliation. Adjusted absorbance is shown for the degree of exfoliation and accounts for sample dilution by multiplying measured absorbance by the value of the dilution. The degree of exfoliation for each spectrum is shown as measured (A), after 30 days (B), and after 60 days (C).

Tauc plot analysis of each exfoliation spectra was completed to determine the optical band gap of the exfoliated nanosheets and shown in ESI Section Spectra analysis, Fig. S6.† These results were compared to the determined optical band gap of the starting protonated LCO powders and discussed in the ESI Section Spectra analysis, Fig. S7.† The optically determined band gaps of each exfoliation condition were related to the degree of acid treatment, with lower band gaps of exfoliated CONs measured for higher acid pre-treatment (3 M HCl, ∼2.2 eV) compared to lower acid pre-treatment (0.1 M HCl, ∼2.4 eV). Each solution of CONs was characterized after 30 and 60 days; notable decreases in peak width (FWHM) and blue shift in the absorption maxima were observed, along with an increase in the band gap. Changes in the absorption profiles for the solutions upon standing can be seen in Fig. 6A–C with a significant drop in absorbance over time (Fig. 7A–C). A decrease in absorption intensity is attributed to the settling of large particles (agglomerated nanosheets), the decrease in peak width is attributed to the narrowing distribution of particle sizes in solution over time, and the blue shift in peak maxima may point to a decreased size of the particles remaining in the solution.

## Conclusions

The exfoliation of LCO was examined as a function of conditions of both steps of exfoliation: proton concentration, NR_4_^+^ ionic size, NR_4_^+^ concentration, and protonated LCO sample aging. Characterization of the supernatant by AFM and UV-Vis absorbance measurements confirmed the presence of two-dimensional CONs. Through the UV-Vis characterization and analysis of multiple reaction conditions, a detailed exfoliation landscape was produced for protonated LCO. Higher acid concentrations used in step 1 yielded protonated powders that gave increased exfoliation yield after step 2, coinciding well with known soft-chemical exfoliation mechanisms. Specifically, 3 M HCl treatment provided the best acid reaction conditions to promote the exfoliation of LCO. Tetraalkylammonium ionic size was another crucial factor for the exfoliation yield, with tetramethylammonium hydroxide providing the most suitable reaction conditions to promote exfoliation. These results elucidated a critical ion size to the interlayer spacing ratio (1.3 : 1) for the exfoliation of LCO. Additionally, the concentration of NR_4_^+^ in solution, the presence of OH^−^ groups, and an aqueous reaction environment were essential for exfoliation to occur. However, exfoliation did not occur for a constant [NR_4_^+^]/[H^+^] ratio across multiple H^+^ concentrations in the powder, challenging the suggestion of such a ratio based on studies that use only one H^+^ concentration in protonated transition metal oxides. Additionally, aging effects of protonated LCO should be considered in any scaling of the process due to a significantly diminished exfoliation yield over time (−56% after 25 days, −75% after 150 days). Finally, the analysis of CONs aging in solution under ambient conditions revealed substantial decreases in concentration (*i.e.*, poor colloidal stability), changes in absorption peak shape, and blue energy shifts and changes in the optically determined band gap.

## Conflicts of interest

The authors declare no competing financial interests.

## Supplementary Material

NA-002-D0NA00755B-s001
