# Supplementary material for: Evaluating the chemical exfoliation of lithium cobalt oxide using UV-Vis spectroscopy
Source: Nanoscale Adv. 2020 Oct 16;2(11):5362–74. doi: 10.1039/d0na00755b (PMC9419208; doi:10.1039/d0na00755b)
Supplement: NA-002-D0NA00755B-s001 [file NA-002-D0NA00755B-s001.pdf]

This journal is © The Royal Society of Chemistry 2020

All raw data, individual spectra analysis, and R code is published in an OSF project https://osf.io/xkme6/ (DOI 10.17605/OSF.IO/XKME6).

SI Section Spectra Analysis

Spectra were labeled for brevity and are as follows,

X-XYZ-XYZ-XX-GXX

where,

Powder (X) – acid treatment (XYZ) – base treatment (XYZ) – days in solution (XX) – measurement group (GXX)

Powder (X):

X = powder ID

- 1 = LCO AR

Acid treatment (XYZ):

X = reagent ID

- 0 = no treatment
- 1 = HCl

Y = molarity

- 0 = no treatment
- 1 = 0.1 M
- 2 = 0.3 M
- 3 = 1 M
- 4 = 3 M

Z = reaction time

- 0 = no treatment
- 1 = 1 day

Base treatment (XYZ):

X = reagent ID

- 0 = no treatment
- 1 = TMAOH
- 2 = TEAOH
- 4 = TBAOH
- 5 = NH_4OH

Y = molarity

- 0 = no treatment
- 1 = 0.014 M
- 2 = 0.044 M
- 3 = 0.14 M
- 4 = 0.44 M
- 5 = 0.0044 M
- 6 = 0.025 M
- 7 = 0.075 M
- 8 = 0.25 M

Z = reaction time

- 0 = no treatment
- 1 = 1 day

Days in solution (XX):

- 00 = Measurement taken immediately after reaction was completed
- 30 = Measurement taken 30 days after reaction was completed
- 60 = Measurement taken 60 days after reaction was completed

Measurement group (GXX):

- G01-G13 = Measurement groups used for identification

Abbreviations:

- NH_4OH = ammonium hydroxide
- TMAOH = tetramethylammonium hydroxide (NMe_4OH)
- TEAOH = tetraethylammonium hydroxide (NEt_4OH)
- TBAOH = tetrabutylammonium hydroxide (NBu_4OH)

All samples were reacted under constant stirring (1000 rpm)

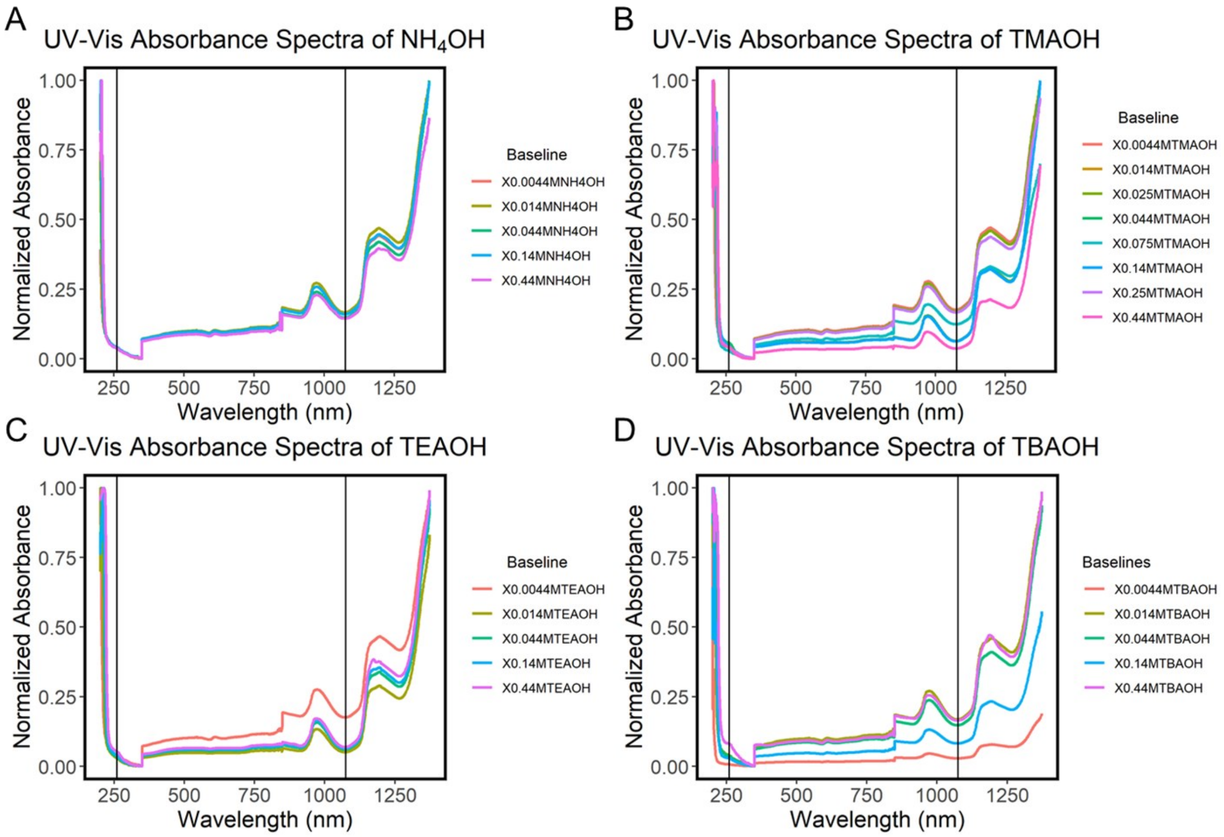

Figure S1: Measured UV-Vis absorbance spectra for NH_4OH (A), TMAOH (B), TEAOH (C), and TBAOH (D) aqueous-based solutions. These measurements were used as baselines and subtracted from each cobalt oxide nanosheet sample. Vertical lines at 260 nm and 1075 nm signify the cut off values for each solvent system.

To properly investigate each spectrum, an appropriate region of the spectrum was determined for reliable data analysis. This process was completed for each aqueous tetraalkylammonium solution (NH_4OH, TMAOH, TEAOH, and TBAOH) at each concentration tested (0.0044 M, 0.014 M, 0.025 M, 0.044 M, 0.075 M, 0.14 M, 0.44 M). Each reagent showed rapid increases in absorbance starting at 245 nm and 1100 nm therefore data collected from each measurement was taken between 260-1075 nm.

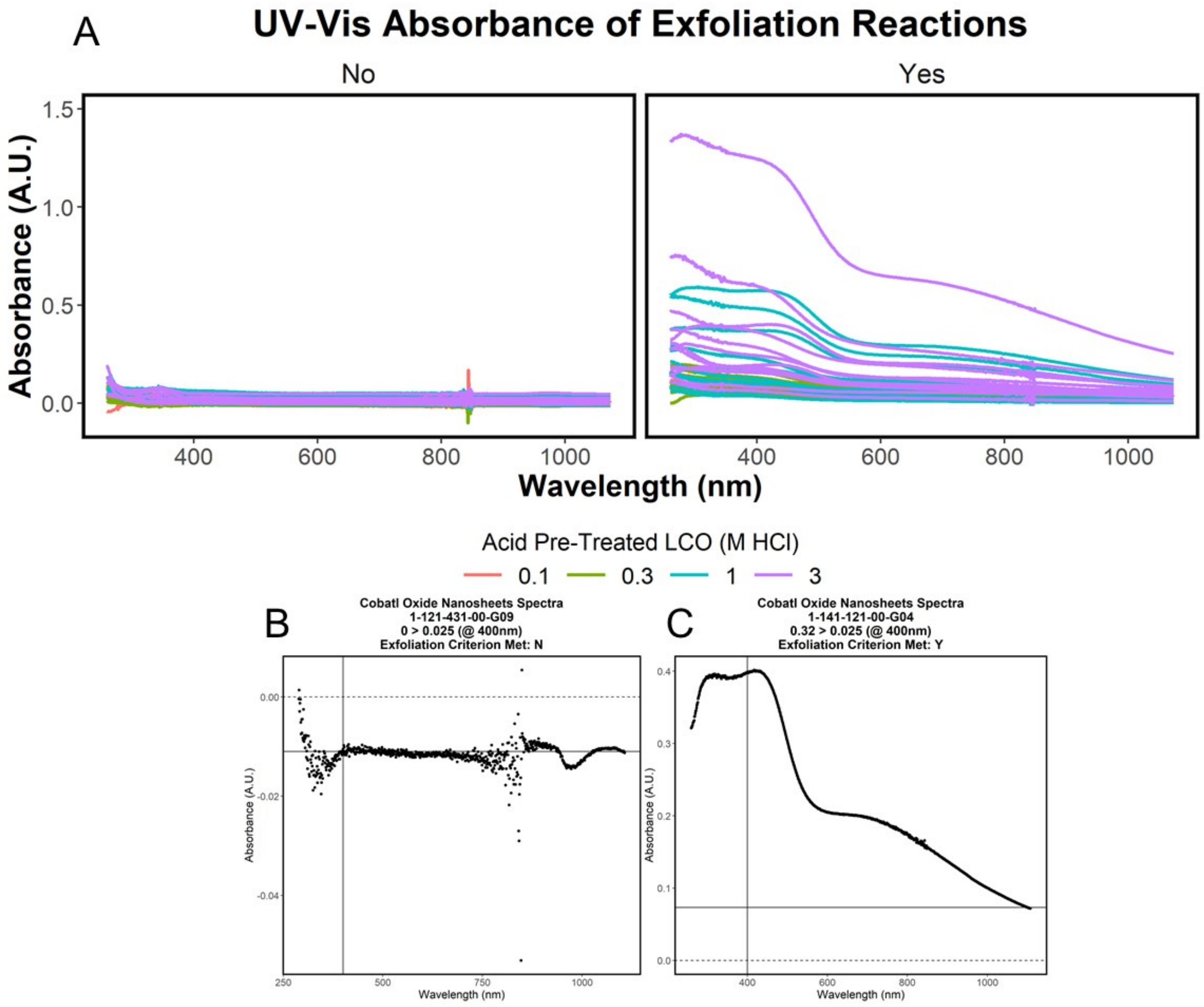

Figure S2: (A) UV-Vis absorbance spectra of each reaction condition after centrifugation to separate unexfoliated particles used to define the exfoliation criterion. The color of each spectrum represents the concentration of the acid used in the acid treatment of LCO signifying varying degrees of proton replacement in the structure, irrespective of base treatment (all base treatments are shown). (B) An example of a reaction condition (LCO, 0.3 M HCl, 0.14 M TBAOH) that did not meet the exfoliation criterion. (C) An example of a reaction condition (LCO, 1 M HCl, 0.044 M TMAOH) that did meet the exfoliation criterion. (B, C) Vertical lines are shown at 400 nm. A solid horizontal line is shown at the absorbance at 1075 nm. A dashed horizontal line is shown at 0 A.U. All powders were reacted for 1 day under constant stirring.

To determine if a reaction solution contained cobalt oxide nanosheets after centrifugation, the absorbance at 1075 nm (a region where little to no absorbance is observed) was subtracted from the absorbance measured at 400 nm to correct for any baseline artifacts. For example, certain reaction conditions produced artifacts such as negative absorbance (e.g. **Figure S2B**) or a baseline absorbance

was observed indicating the presence of thicker particles with featureless absorbance (e.g. **Figure S3b, S3d**). If the corrected absorbance value was greater than 0.025 A.U., the solution was classified as having a measurable concentration of cobalt oxide nanosheets in solution. Scattering effects are typically a function of particle size and wavelength, with scattering effects increasing significantly as wavelength decreases for large particles. These effects were not observed in these measurements as a result of the use of an integrating sphere.

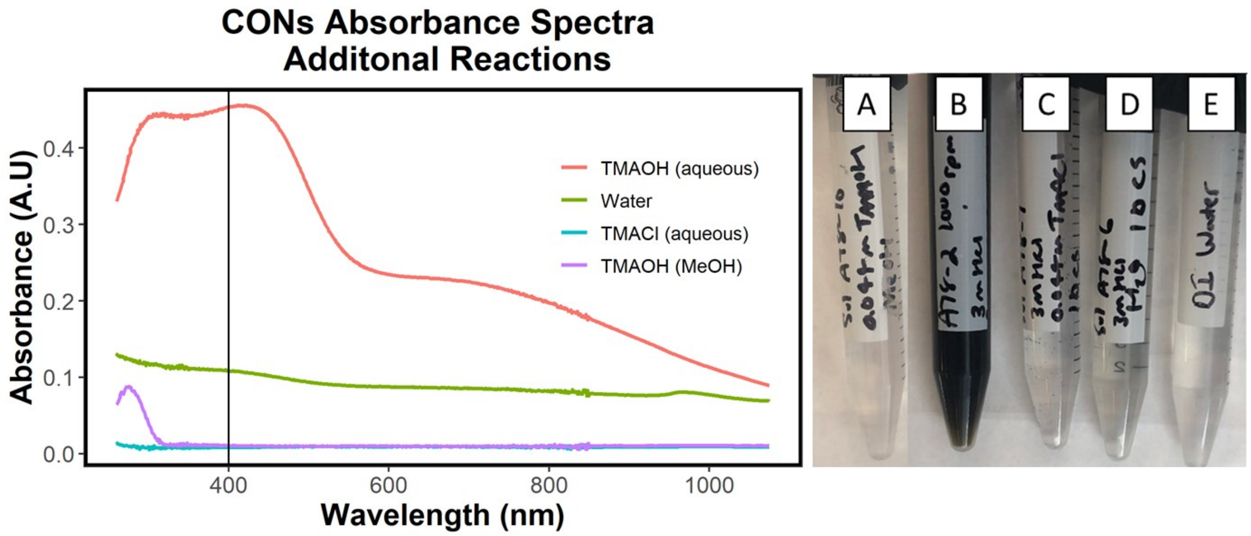

Figure S3: Measured UV-Vis absorbance spectra and optical image of 3 M HCl protic lithium cobalt oxide exfoliated using 0.044 M TMAOH aqueous (orange, B), water (green, D), 0.044 M TMACl aqueous (blue, C), and 0.044 M TMAOH methanol (purple, A). Shown is DI water for comparison purposes (E).

UV-Vis absorbance spectra of 3 M HCl protic LCO powder after various reaction conditions show distinct differences in absorbance profiles. 0.044 M TMAOH shows a distinct 3 broad peak absorbance spectra, whereas exfoliation using DI water, 0.044 M TMACl, and 0.044 M TMAOH (in MeOH) do not show characteristic cobalt oxide nanosheet absorbance peaks. A broad baseline absorbance is observed for the water exfoliated solution and is likely due to the presence of a small concentration of thick LCO suspended in solution. Optical images of solutions after various reaction conditions and centrifugation are also shown. The reaction conditions tested show a stark difference in color and turbidity with only 0.044 M TMAOH (aqueous) producing a stable suspension of cobalt oxide nanosheets, while the others remain clear and colorless after centrifugation.

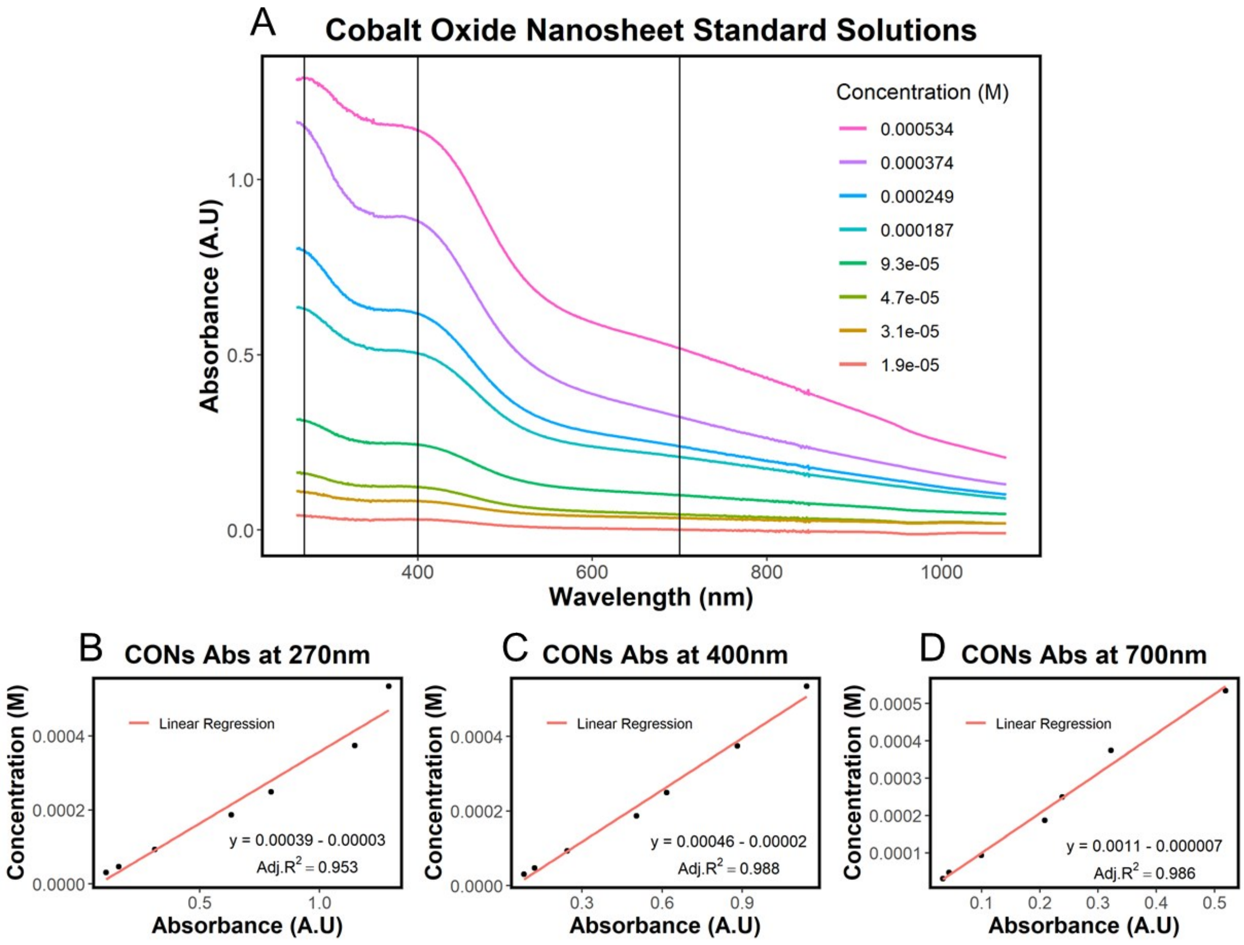

Figure S4: (A) Measured UV-Vis absorbance spectra of cobalt oxide nanosheet standard solutions colored by concentration. Vertical lines are shown at 270 nm, 400 nm, and 700 nm. (B) A calibration curve and linear regression made from the absorbance at 270 nm. (C) A calibration curve and linear regression made from the absorbance at 400 nm. (D) A calibration curve and linear regression made from the absorbance at 700 nm.

To determine an appropriate and reliable wavelength to compare each spectrum, a solution of cobalt oxide nanosheets with a known concentration (0.0187 mol CoO_2 / L) was diluted to make multiple standard solutions and measured using UV-Vis absorbance spectroscopy. The measured UV-Vis absorbance for each solution was taken at 270 nm, 400 nm, and 700 nm and plotted as a function of concentration to produce calibration curves for each wavelength of interest. Linear regression was completed for each wavelength and determined that 400 nm (R^2 = 0.988) was the most suitable for

comparison purposes. The absorption coefficient at 400 nm (2640 $L \cdot mol^{-1} \cdot cm^{-1}$) was calculated using Beer's Lambert law described in equation 1.

$$A = \alpha C l \qquad (1)$$

Where A is the measured absorbance, α is the molar absorption coefficient of the sample, C is the concentration of the sample, and l is the path length light travels through the sample.

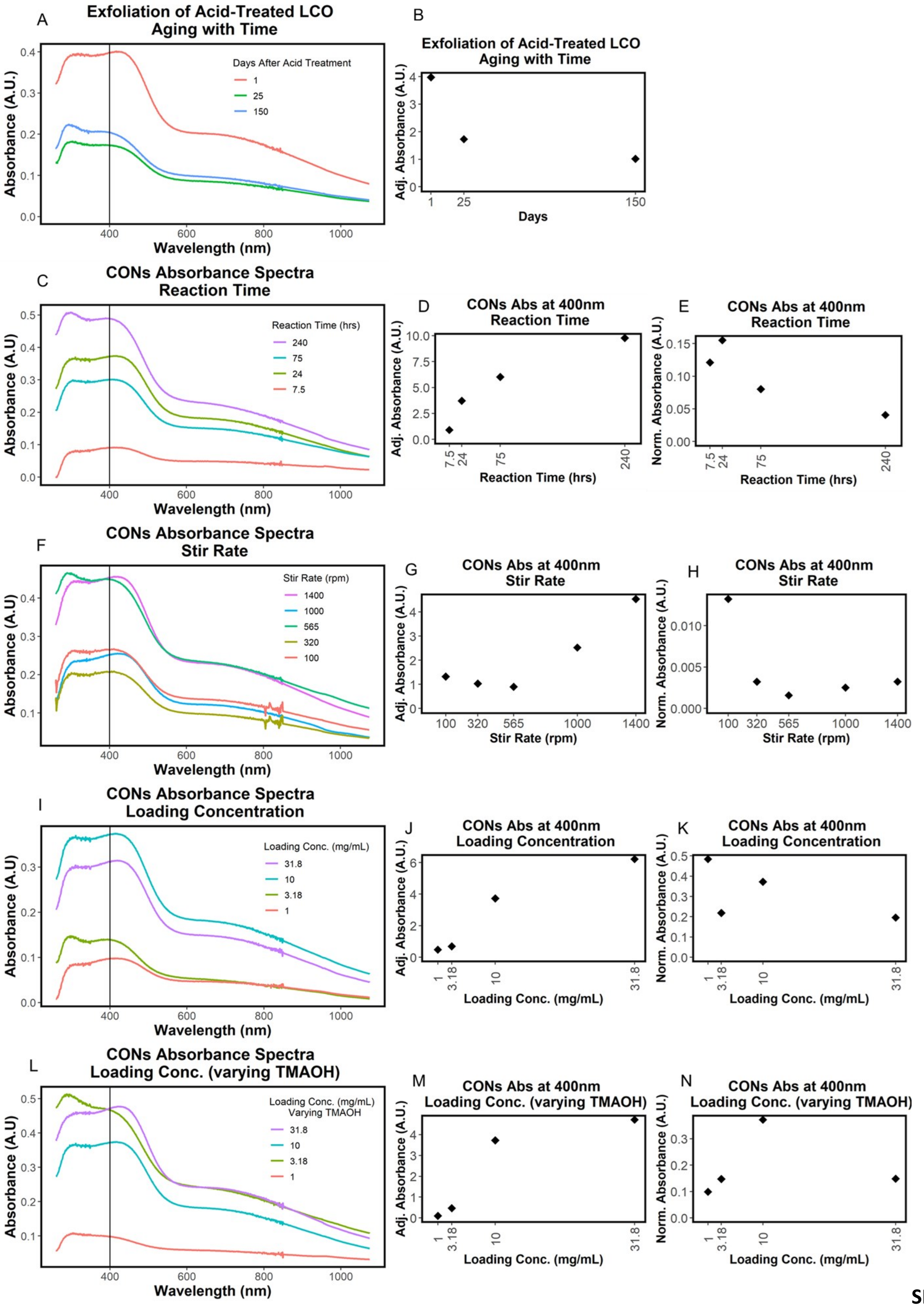
A
Exfoliation of Acid-Treated LCO
Aging with Time
Days After Acid Treatment
1
25
150
Absorbance (A.U.)
0.0
0.1
0.2
0.3
0.4
400
600
800
1000
Wavelength (nm)
B
Exfoliation of Acid-Treated LCO
Aging with Time
Adj. Absorbance (A.U.)
0
1
2
3
4
1
25
150
Days
C
CONs Absorbance Spectra
Reaction Time
Reaction Time (hrs)
240
75
24
7.5
Absorbance (A.U)
0.0
0.1
0.2
0.3
0.4
0.5
400
600
800
1000
Wavelength (nm)
D
CONs Abs at 400nm
Reaction Time
Adj. Absorbance (A.U.)
0.0
2.5
5.0
7.5
10.0
7.5
24
75
240
Reaction Time (hrs)
E
CONs Abs at 400nm
Reaction Time
Norm. Absorbance (A.U.)
0.00
0.05
0.10
0.15
7.5
24
75
240
Reaction Time (hrs)
F
CONs Absorbance Spectra
Stir Rate
Stir Rate (rpm)
1400
1000
565
320
100
Absorbance (A.U)
0.1
0.2
0.3
0.4
400
600
800
1000
Wavelength (nm)
G
CONs Abs at 400nm
Stir Rate
Adj. Absorbance (A.U.)
0
1
2
3
4
100
320
565
1000
1400
Stir Rate (rpm)
H
CONs Abs at 400nm
Stir Rate
Norm. Absorbance (A.U.)
0.000
0.005
0.010
100
320
565
1000
1400
Stir Rate (rpm)
I
CONs Absorbance Spectra
Loading Concentration
Loading Conc. (mg/mL)
31.8
10
3.18
1
Absorbance (A.U)
0.0
0.1
0.2
0.3
400
600
800
1000
Wavelength (nm)
J
CONs Abs at 400nm
Loading Concentration
Adj. Absorbance (A.U.)
0
2
4
6
1
3.18
10
31.8
Loading Conc. (mg/mL)
K
CONs Abs at 400nm
Loading Concentration
Norm. Absorbance (A.U.)
0.0
0.1
0.2
0.3
0.4
0.5
1
3.18
10
31.8
Loading Conc. (mg/mL)
L
CONs Absorbance Spectra
Loading Conc. (varying TMAOH)
Loading Conc. (mg/mL)
Varying TMAOH
31.8
10
3.18
1
Absorbance (A.U)
0.1
0.2
0.3
0.4
0.5
400
600
800
1000
Wavelength (nm)
M
CONs Abs at 400nm
Loading Conc. (varying TMAOH)
Adj. Absorbance (A.U.)
0
1
2
3
4
1
3.18
10
31.8
Loading Conc. (mg/mL)
N
CONs Abs at 400nm
Loading Conc. (varying TMAOH)
Norm. Absorbance (A.U.)
0.0
0.1
0.2
0.3
1
3.18
10
31.8
Loading Conc. (mg/mL)

SI

Figure S5: Unless otherwise stated, all reactions were completed with 3 M HCl treated LCO, 24 hrs, 10 mg/mL, and 1000 rpm. (A) Measured UV-Vis absorbance spectra of cobalt oxide nanosheet solutions exfoliated from protic lithium cobalt oxide one day (orange), 25 days (green), and 150 days (blue) after the completion of acid treatment. (C) Measured UV-Vis absorbance spectra of cobalt oxide nanosheet solutions exfoliated from protic lithium cobalt oxide after reacting for 7.5 hours (orange), 24 hours (green), and 75 hours (blue), and 240 hours (purple). (F) Measured UV-Vis absorbance spectra of cobalt oxide nanosheet solutions exfoliated from protic lithium cobalt oxide reacting at 100 rpm (orange), 320 rpm (olive), and 565 rpm (turquoise), 1000 rpm (blue), and 1400 rpm (magenta). (I) Measured UV-Vis absorbance spectra of cobalt oxide nanosheet solutions exfoliated from protic lithium cobalt oxide after reacting with concentrations of 1 mg/mL (orange), 3.18 mg/mL (green), 10 mg/mL (blue), and 31.8 mg/mL (purple) with 0.044 M TMAOH (L) Measured UV-Vis absorbance spectra of cobalt oxide nanosheet solutions exfoliated from protic lithium cobalt oxide after reacting with 1 mg/mL - 0.0044 M TMAOH (orange), 3.18 mg/mL - 0.014 M TMAOH (green), 10 mg/mL - 0.044 M TMAOH (blue), and 31.8 mg/mL - 0.14 M TMAOH (purple). TMAOH was varied for each reaction such that a $[TMA^+]/[H^+]$ ratio was held constant. (B, D, G, J, M) Adjusted absorbance as a function of aging time after completion of acid treatment (B), reaction time (D), stir rate (G), loading concentration (J), and loading concentration of varying TMAOH providing constant $[TMA^+]/[H^+]$ (M). Adjusted absorbance accounts for sample dilution by multiplying measured absorbance with the value of the dilution. (E, H, K, N) Normalized absorbance was plotted as a function of reaction time (E), stir rate (H), loading concentration (K), and loading concentration of varying TMAOH (N). Normalized absorbance is calculated by dividing the x-axis value by the adjusted absorbance.

Additional reaction conditions after 3M HCl treatment for protic lithium cobalt oxide were investigated. First, a notable decrease in exfoliation rate was observed by reacting the same protic LCO powder 1 day, 25 days, and 150 days after the completion of acid treatment. This is likely due to diffusion in the powder, causing a more stable protic structure over time. Second, reaction time was considered as a reaction parameter. The rate of reaction follows a logarithmic trend with increased time. Normalized absorbance is calculated by dividing the x-axis value by the adjusted absorbance. For example, the absorbance measured for different reaction times (e.g. 7.5 hrs, 24 hrs, 75 hrs, 240 hrs) was divided by the reaction time for that condition (Adj. Abs / Rxn time = Normalized Abs.). Normalization showed that 1 day treatment provided the maximal return of exfoliation rate vs. time. Third, the reaction stir rate was considered for its effects on the rate of exfoliation. Stir rates were tested from 100 rpm (the lowest setting) to 1400 rpm (the highest setting). It was observed that at low stir rates there is no dependence of exfoliation rate on stir rate. As the stir rate was increased to 1000 rpm and 1400 rpm, an increase in the exfoliation rate was observed. This is likely due to the additional mechanical force

applied at higher stir rates aiding with exfoliation. Normalization shows that 100 rpm returned the maximal exfoliation rate by stir rate. All other stir rates level out and are not significant as observed through normalization. Fourth, loading concentration was considered for its effects on the exfoliation rate. 0.044 M TMAOH solutions were reacted with multiple concentrations of 3 M HCl protic LCO powder. A positive correlation between loading and exfoliation rate is observed. Normalization to loading concentration shows that lower loading concentrations provided the highest rates of exfoliation and are expected. Fifth, loading concentration was investigated while keeping a constant TMA^+/H^+ ratio. To do so, if the loading concentration was lower 10x (e.g. 1 mg/mL), the TMAOH concentration was lower accordingly (0.0044 M TMAOH). This was completed for each sample and was used to examine the effect of a constant TMA^+/H^+ ratio by changing the powder concentration in solution, instead of the H^+ concentration in the powder. When compared to the loading concentration with a constant TMAOH, these reaction conditions did not perform as well and showed considerably lower exfoliation rates before and after normalization. It was determined that if loading concentration is to be changed, TMAOH concentration should remain constant, as opposed to a constant TMA^+/H^+ ratio.

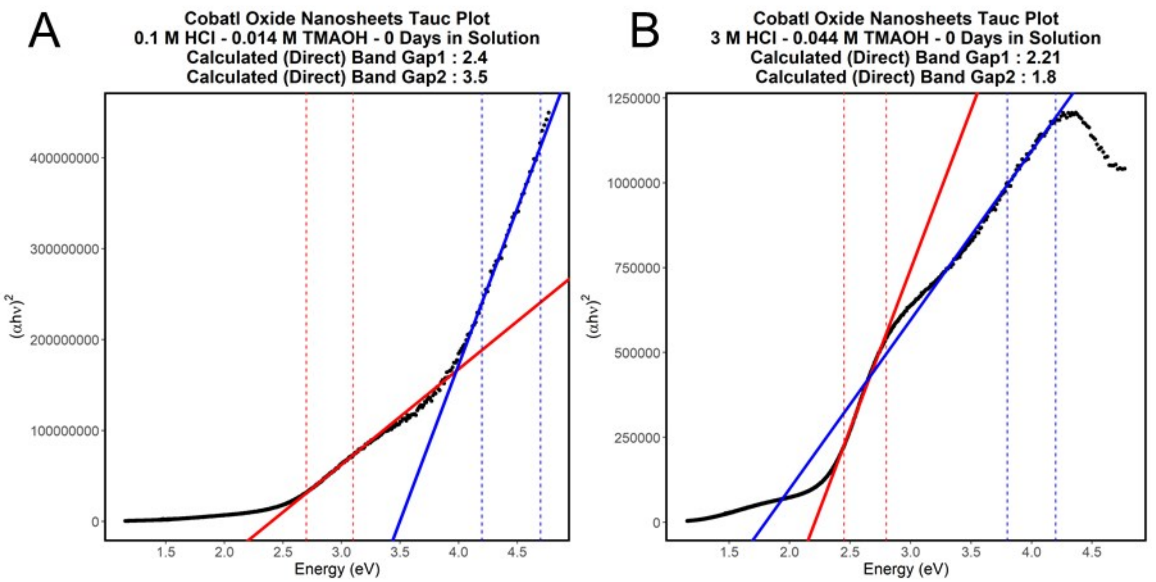

Figure S6: Calculated Tauc plots ($(\alpha h\nu)^2$ vs. hν) of 0.1 M HCl + 0.014 M TMAOH cobalt oxide nanosheet solution (A) and 3 M HCl + 0.044 M TMAOH cobalt oxide nanosheet solution (B). Linear extrapolated regions are shown for the calculated optical band gap 1 (red) and calculated optical band gap 2 (blue). Dashed lines represent the region of fit for each optical band gap.

Optical band gaps for each sample that met the exfoliation criterion were calculated using Tauc plot analysis. UV-Vis absorbance spectra were converted to the absorption coefficient as a function of wavelength using the Beer-Lambert Law described in equation 1. Next, a Tauc plot was made for each sample using equation 2. Where h is Planck's constant, ν is frequency, α is the absorption coefficient of the sample, A is the proportionality constant, E_g is the optically determined band gap, and n is equal to 0.5, 2 for indirect and direct band gaps, respectively.

$$(\alpha h\nu)^n = A(h\nu - E_g) \qquad (2)$$

The transformation of UV-Vis absorbance data into Tauc plots provides a suitable method for comparing the electronic properties of the cobalt oxide nanosheets after each exfoliation reaction. Optical band gap modeling of UV-Vis absorbance data has been completed on many types of materials, such as nanomaterials, and transition metal oxides but must assume the nature of the band gap transition (i.e. direct or indirect). It was assumed that cobalt oxide nanosheets have direct band gaps. Tauc plot

analysis of cobalt oxide nanosheet solutions provided two linear regions of interest, allowing for the calculation of two band gaps, band gap 1 and band gap 2. Though it is not likely for two band gaps to exist in this system, these two linear regions may represent the intrinsic band gap, excitons, or the presence of two material classes (i.e. small particles vs. large particles, or Co^{3+} vs. Co^{4+}). Linear regression on regions of the Tauc transformed ($(\alpha h\nu)^2$) data increasing linearly with energy was completed. The linear fit (red for band gap 1, blue for band gap 2) was extrapolated to the x-axis where the optical band gap for each region was determined.

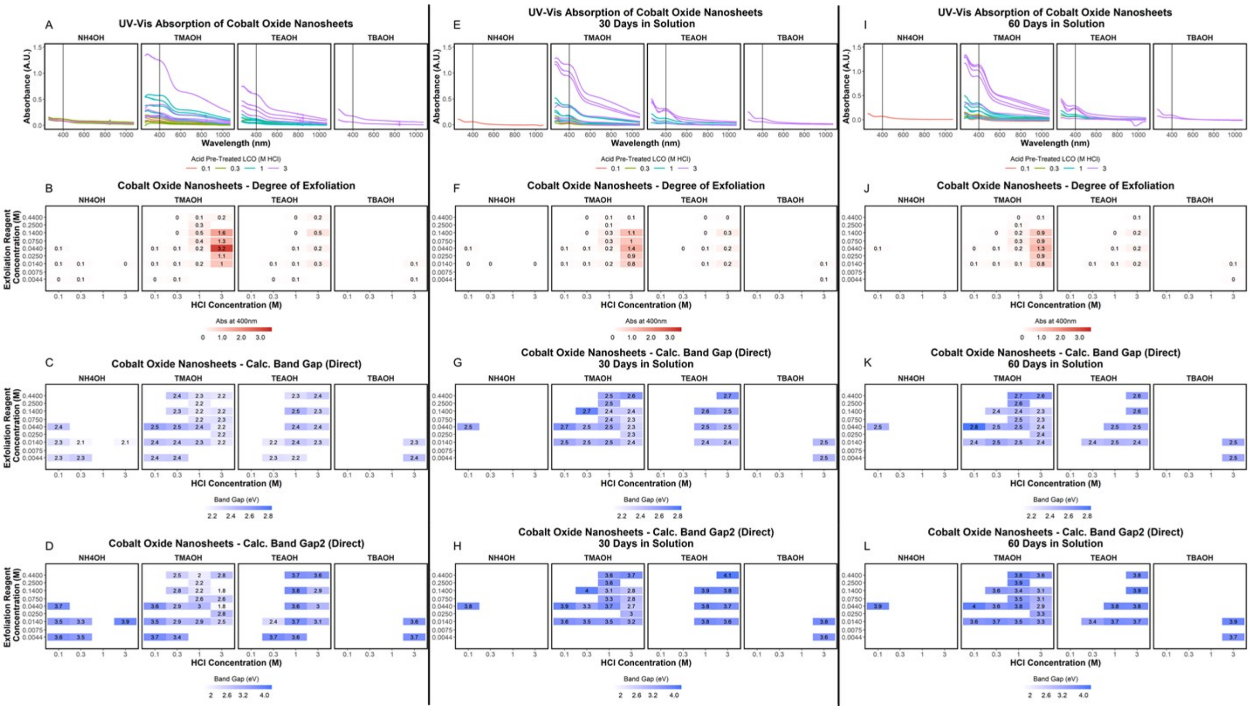

Figure S7: (A, E, I) Measured UV-Vis absorbance spectra of exfoliated cobalt oxide nanosheet solutions separated by the ionic radius of the exfoliation reagent used. The color of each spectrum represents the concentration of the acid of pre-treated LCO signifying varying degrees of proton replacement in the structure. A vertical line is shown at 400 nm; the absorbance at this wavelength was taken as the value for the degree of exfoliation. Spectra are shown as measured (A), after 30 days (E), and after 60 days (I). (B, F, J) The degree of exfoliation for each spectrum was plotted in a 2D array with pre-treated acid powder (0.1 M HCl, 0.3 M HCl, 1 M HCl, 3 M HCl) as the x-axis, the concentration of the exfoliation reagent (0.0044 M, 0.0075 M, 0.014 M, 0.025 M, 0.044 M, 0.075 M, 0.14 M, 0.25 M, 0.44 M) as the y-axis, and separated by the ionic radius of the exfoliation reagent used. A scale bar at the bottom of the plot shows the variation of color corresponding to the degree of exfoliation. Adjusted absorbance is shown for the degree of exfoliation and accounts for sample dilution by multiplying measured absorbance by the value of the dilution. The degree of exfoliation for each spectrum is shown as measured (B), after 30 days (F), and after 60 days (J). (C, G, K) The optical band gap 1 for each spectrum was plotted in a 2D array with pre-treated acid powder (0.1 M HCl, 0.3 M HCl, 1 M HCl, 3 M HCl) as the x-axis, the concentration of the exfoliation reagent (0.0044 M, 0.0075 M, 0.014 M, 0.025 M, 0.044 M, 0.075 M, 0.14 M, 0.25 M, 0.44 M) as the y-axis, and separated by the ionic radius of the exfoliation reagent used. A scale bar at the bottom of the plot shows the variation of color corresponding to the value of the optical band gap. The optical band gap 1 for each spectrum is shown as measured (C), after 30 days (G), and after 60 days (K). (D, H, L) The optical band gap 2 for each spectrum was plotted in a 2D array with pre-treated acid powder (0.1 M HCl, 0.3 M HCl, 1 M HCl, 3 M HCl) as the x-axis, the concentration of the exfoliation reagent (0.0044 M, 0.0075 M, 0.014 M, 0.025 M, 0.044 M, 0.075 M, 0.14 M, 0.25 M, 0.44 M) as the y-axis, and separated by the ionic radius of the exfoliation reagent used. A scale bar at the bottom of the plot shows the variation of color corresponding to the value of the optical band gap. The optical band gap 2 for each spectrum is shown as measured (D), after 30 days (H), and after 60 days (L).

A summary of the optically determined direct band gap for each exfoliation condition is shown along with changes to the calculated band gap over time. Conditions that produced exfoliated cobalt oxide nanosheets in solution were plotted with exfoliation reagent concentration (y-axis), HCl concentration (x-axis), and exfoliation reagents identity (each plot). Calculated band gaps for each spectrum fell within the range of 2.15 – 2.6 eV for the first region of interest (band gap 1). Calculated band gaps for each spectrum fell within the range of 2.0-3.7 eV for the second region of interest (band gap 2). Of note, it was generally observed for each exfoliation reagent that higher acid treatments resulted in lower band gaps of exfoliated cobalt oxide nanosheets. Each sample had an observable blue shift with aging, with increases in both band gaps measured (approximately +0.1-0.9 eV) after 30 and 60 days for nearly all samples.

SI Section Acid Treated Powders

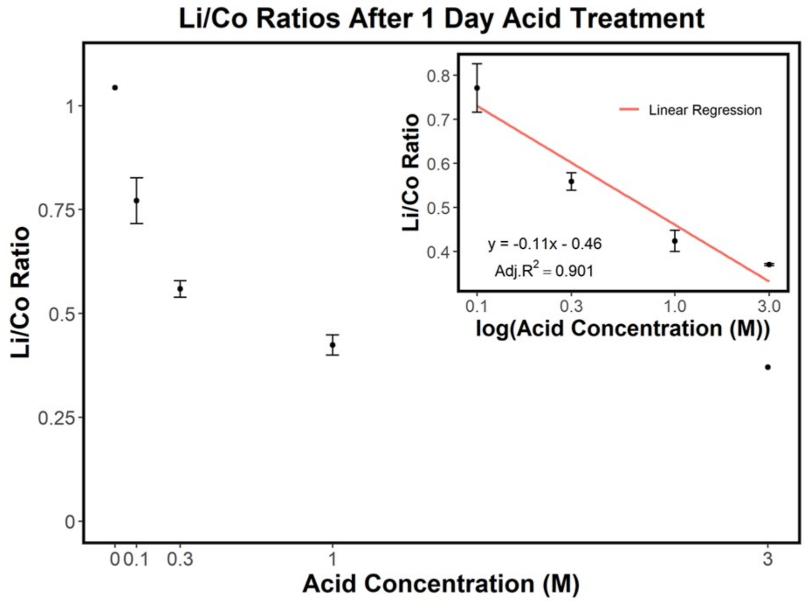

Figure S8: Li/Co ratios after various acid treatments of $LiCoO_2$ measured using ICP-MS. Uncertainties are shown for all samples except LCO AR (i.e. 0 M acid concentration). A logarithmic dependence is shown and plotted in the upper right corner excluding LCO AR. An adjusted R^2 of 0.90 was determined for the linear regression and is shown. All powders were reacted for 1 day under constant stirring.

The measured lithium to cobalt ratio of lithium cobalt oxide as received and after 0.1 M, 0.3 M, 1 M, and 3 M HCl acid treatments using ICP-MS characterization are plotted with uncertainties reported as 1 standard deviation. Uncertainty was not determined for lithium cobalt oxide as received. A logarithmic relationship between acid concentration and Li/Co ratio is observed and shown in the upper panel of the plot. Due to the complete dissolution of LCO at high acid concentrations (>10 M) under the same reaction conditions, no data points were reported at these conditions.

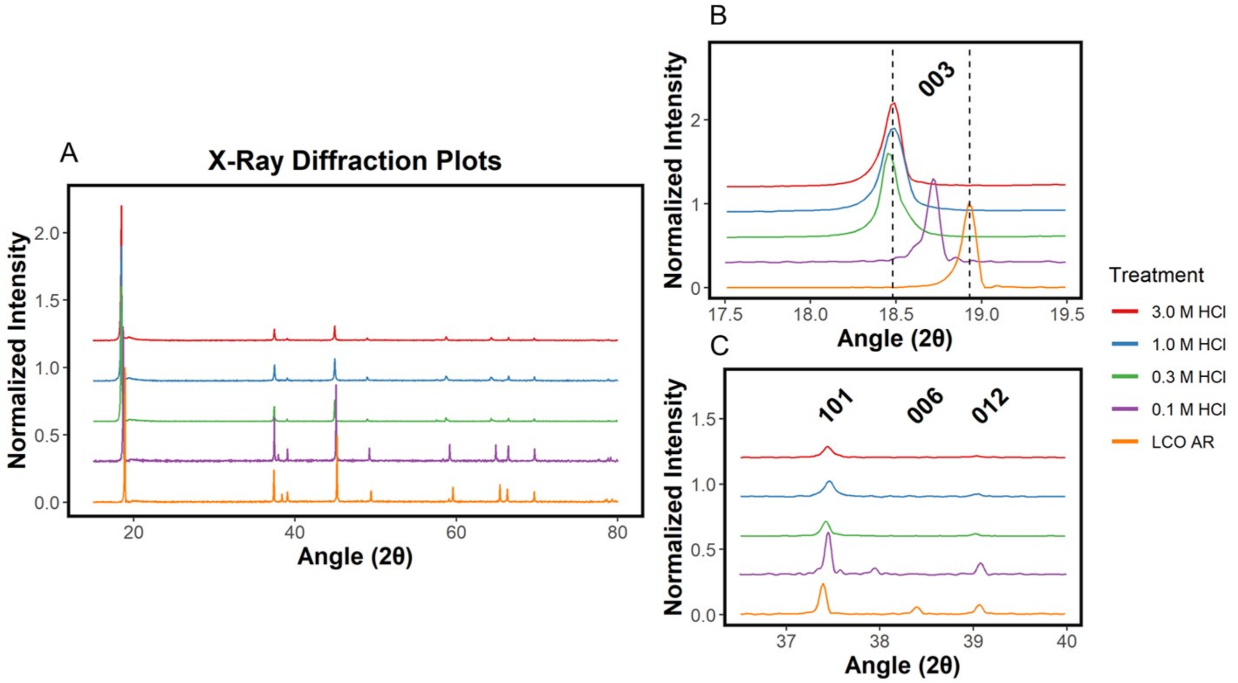

Figure S9: (A) Normalized intensity X-ray Diffraction plots of LCO AR, 0.1 M HC, 0.3 M HCl, 1 M HCl, and 3 M HCl acid-treated LCO are shown from 15-80°2θ, (B) 17.5-19.5°2θ, and (C) 36.5-40°2θ. Selected diffraction peaks are shown for 003 (B), and 101, 006, and 012 (C). All powders were reacted for 1 day under constant stirring.

Normalized intensity of X-ray diffraction for each lithium cobalt oxide as received and after 0.1 M, 0.3 M, 1 M, and 3 M HCl acid treatment are shown. Noticeable decreases in peak intensity ratios were observed for all peaks in relation to the 003 reflection. No peaks indicating a phase change or a second phase formation were observed. This decrease in intensity is likely due to preferential etching at certain crystallographic planes and an increase in preference of the powder to orient on the surface of the zero-background holder due to the change in its morphology. A shift of 003, 006 reflections to lower angles demonstrated an expansion of the c-lattice parameter.

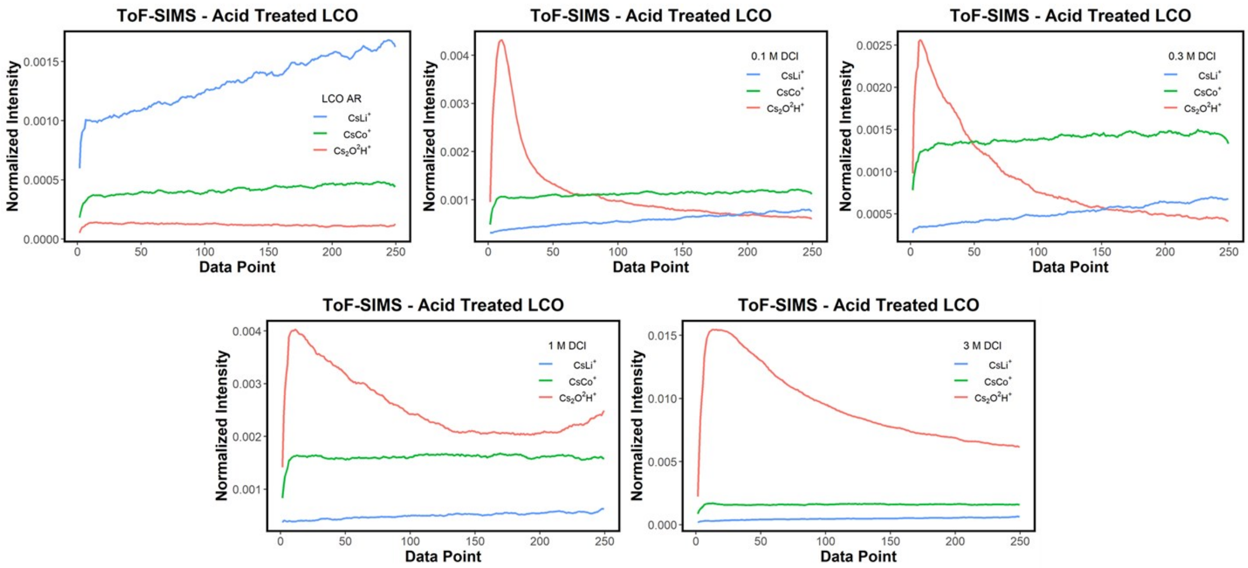

Figure S10: Normalized intensity ToF SIMS plots as a function of data points (sputtering depth) of deuterium (orange), lithium (blue), and cobalt (green) containing matrix components for LCO AR and LCO after 0.1 M, 0.3 M, 1 M, and 3 M deuterium chloride treatment. Powders were reacted for 1 day under constant stirring.

Shown is the normalized (to total count) intensity ToF SIMS measurements on lithium cobalt oxide as received and after 0.1 M, 0.3 M, 1 M, and 3 M DCl deuterated acid treatment for $CsLi^+$, $CsCo^+$, $Cs_2O^2H^+$. To compare the relative intensity of deuterium in each sample, the normalized intensity over the measured area (0-250 collected data points) was averaged for each species. Since multiple particles were measured by the ToF SIMS instrument for each sample, local fluctuations in concentration within individual particles were averaged with this method. Matrix effects are seen for all species but their influence in conclusions was reduced when analyzing MCs^+ clusters. Due to the relatively stable intensities of Li and Co containing species for all samples across all data points measured, it is unlikely this method observed any core-shell elemental distribution in the powders and likely due to the analysis of multiple particles at once for each sample tested.

A positive high mass resolution depth profile was performed using a TOF-SIMS NCS instrument at the Shared Equipment Authority from Rice University. A bunched 30 keV Bi_3^+ ions (with a measured current of 0.15 pA) was used as a primary probe for depth profiling the field of view of 100 × 100 µm^2,

with a raster of 64 by 64 pixels, and then the sputtering was performed using Cs^+ ions at 2 keV with a typical current around 135 nA, rastered area 300 × 300 μm^2. The beams were operated in non-interlaced mode, alternating 3 analysis cycles and 5s sputtering cycles followed by a pause of 3s for the charge compensation with an electron flood gun. Again an adjustment of the charge effects has been operated using a surface potential of 5V and an extraction bias of 20V. During the depth profiling, the cycle time was fixed to 90 µs (corresponding to m/z = 0 – 738 a.m.u mass range). The MCs_n^+ (n=1, 2) depth profiling is a useful method, mainly applied to quantify the alloys but also to identify any ion compounds. The cesium primary beam is used for sputtering during the depth profile and permits to detect MCs^+ or MCs_2^+ cluster ions where M is the element of interest combined with one or two Cs atoms. The advantages of following MCs^+ and MCs_2^+ ions during SIMS analysis include the reduction of matrix effects and the possibility of detecting the compounds from both electronegative and electropositive elements.[1–3]

Table S1: A summary of the averaged normalized intensity of each analyte measured using ToF SIMS for LCO AR and deuterated acid-treated samples. The normalized intensity was averaged for each species and samples across the measured region (data points 0-250).

	Mass (u)	LCO_AR	0.1 M DCl	0.3 M DCl	1 M DCl	3 M DCl
$^{2}H^{+}$	2.0136	1.19E-07	1.41E-06	8.48E-07	3.22E-06	1.01E-05
H_2^{+}	2.016	4.77E-06	2.54E-06	2.48E-06	3.17E-06	1.62E-06
Li^{+}	7.0168	0.104659	0.02289	0.019333	0.016281	0.016757
Na^{+}	22.9905	0.001033	0.000863	0.000511	0.000197	0.000233
Mg^{+}	23.9838	2.35E-05	4.97E-05	7.06E-05	6.71E-05	7.52E-05
Ca^{+}	39.96	3.25E-05	1.45E-05	3.65E-05	2.36E-05	1.25E-05
Ti^{+}	47.9434	6.01E-06	3.66E-05	7.34E-06	7.49E-06	6.95E-06
Co^{+}	58.9326	0.000131	0.000515	0.000705	0.000729	0.000749
Cs^{+}	132.9258	0.613272	0.706587	0.725923	0.729583	0.744661
$CsLi^{+}$	139.9174	0.001311	0.000578	0.000511	0.000497	0.000466
$CsNa^{+}$	155.9249	0.004956	0.001371	0.001243	0.001121	0.001182
$CsMg^{+}$	156.9425	0.010742	0.001566	0.000908	0.000776	0.000586
$CsCo^{+}$	191.8441	0.000416	0.001116	0.001388	0.001609	0.001595
$CsCoO^{+}$	207.8245	0.000262	0.000626	0.000828	0.000903	0.001044
Cs_2^{+}	265.8173	0.01087	0.026496	0.028921	0.032373	0.027588
Cs_2H^{+}	266.8398	0.000991	0.002028	0.002079	0.00245	0.001023
Cs_2O^{+}	281.8049	0.020101	0.019256	0.023278	0.020501	0.029097
$^{2}HCs_2^{+}$	281.8049	1.36E-06	0.019256	0.023278	0.020501	0.029097
Cs_2OH^{+}	282.8385	0.108275	0.071446	0.059347	0.057275	0.045323
$^{2}HOCs_2^{+}$	283.8446	0.000122	0.001208	0.000923	0.002578	0.009464
Cs_2F^{+}	284.8294	0.00029	0.006877	0.006601	0.000617	0.000507

A summary of the ToF SIMS averaged normalized intensity for each species measured. The normalized intensity was averaged for each species and samples across all data points measured (data points 0-250).

Table S2: A summary of calculations used for ToF SIMS measurements for each lithium cobalt oxide and deuterated acid-treated samples.

	LCO AR	0.1 M DCl	0.3 M DCl	1 M DCl	3 M DCl
Li containing	0.131%	0.058%	0.051%	0.050%	0.047%
Co containing	0.042%	0.112%	0.139%	0.161%	0.159%
D containing	0.012%	0.121%	0.092%	0.258%	0.946%
D containing adjusted	0.000%	0.109%	0.080%	0.246%	0.934%
Li/Co ratio (SIMS)	3.15	0.52	0.37	0.31	0.29
Li/Co ratio (ICP)	1.04	0.73	0.54	0.43	0.37
scaling factor	0.33	1.41	1.47	1.39	1.27
D/Li ratio (SIMS)	0.00	1.88	1.57	4.94	20.06
D/Li ratio (full adj)	0.00	2.65	2.30	6.87	25.42
D/Co ratio (SIMS)	0.00	0.97	0.58	1.53	5.86
D/Co ratio (full adj)	0.00	1.37	0.85	2.12	7.42

Li containing ($CsLi^+$), Cs containing ($CsCo^+$), and deuterium containing ($Cs_2O^2H^+$) normalized values were averaged from 0-250 data points and used for calculating relative D/Li and D/Co ratios. Due to the presence of low intensity of deuterium containing species in LCO AR, this value was subtracted as a baseline from each sample and is attributed to noise within the ToF SIMS measurement. A scaling factor was then calculated for each sample relating to the Li/Co ratio determined by ToF SIMS and ICP-MS measurements on the same powder. Differences in scaling factors for each sample are attributed to varying bonding chemistry associated with the different acid treatments tested. Adjusted D/Li and D/Co ratios were determined for each sample using the scaling factor determined for each sample. It is assumed that Li/Co scaling for each sample allows for the measurement of D/Li and D/Co due to

constant scaling for all species. It was observed that with high acid treatment, a high concentration of deuterium containing species was measured.

Table S3: A summary of the a- and c- lattice parameters for LCO before and after various treatments determined by XRD characterization and Rietveld refinement. 3 M HCl LCO - 1 corresponds to the first sample measured over time. 3 M HCl LCO – 2 corresponds to the second sample measured before and after drying. 003 d-spacing is indicative of the interlayer spacing. Samples were measured after drying and being submerged in water. All submerged samples were measured in a wet/slurry state. All powders were reacted for 1 day under constant stirring.

Sample	a-lattice parameter	c-lattice parameter	003 d-spacing
LCO	2.819	14.07	4.687
3 M HCl LiCoO2 -1 dried	2.811	14.40	4.799
3 M HCl LiCoO2 -1 dried, measured after 167 days	2.811	14.38	4.794
3 M HCl LiCoO2 – 1 soaked in water for 1 month	2.819	14.39	4.797
3 M HCl LiCoO2 – 2 dried	2.811	14.37	4.791
3 M HCl LiCoO2 – 2 remained submerged in water	2.812	14.38	4.793

Two 3 M HCl treated samples were examined using XRD and Rietveld refinement to investigate the effect time and water submersion have on the measured lattice parameters. Sample 3 M HCl $LiCoO_2$ – 1 was measured dried and measured again after 167 days with minimal changes in lattice parameters (-0.01 Å). This sample was also measured after being submerged in water for 1 month with a partial change of lattice parameters back to when the sample was initially measured. Sample 3 M HCl $LiCoO_2$ – 2 was a repeat 3 M HCl acid-treated sample and was split into two samples, one dried and one remaining wet through constant water submersion. It is observed that there are no noticeable changes in swelling (i.e. hydration) occur for the wet sample when compared to the dried sample. Therefore, there are no water molecules present in the interlayer after acid treatment.
